# Standard Addition as a Method for Quantitative Mass Spectrometry Imaging

**DOI:** 10.1021/acs.analchem.5c00549

**Published:** 2025-05-27

**Authors:** Lucie Davidová, Ingela Lanekoff

**Affiliations:** † Department of Chemistry—BMC, 8097 Uppsala University, Box 576, 751 23 Uppsala, Sweden; ‡ Center of Excellence for the Chemical Mechanisms of Life, Uppsala University, 751 23 Uppsala, Sweden

## Abstract

In mass spectrometry imaging (MSI), analytes are desorbed and ionized directly from a complex and unique chemical microenvironment in each pixel, which makes their quantification challenging. Matrix effects have been addressed by the use of isotopically labeled internal standards (IS), either included in the solvent or sprayed over the tissue section, for pixel-by-pixel relative quantification. However, in addition to requiring preselection, isotopically labeled IS may be costly or unavailable. Here, we introduce a novel approach for quantification in MSI, based on the standard addition method. We report a workflow for both acquiring and processing quantitative data. Furthermore, we compare the detected concentrations obtained by standard addition to the detected concentrations obtained using both IS quantification and external calibration. Finally, we show the applicability of using molecules extracted from tissue as an easily accessible standard mixture for standard addition quantification in MSI. The possibility of using analytical standards and readily available endogenous analytes as a source of calibration standards makes our standard addition-based quantitative approach cost-effective, accessible, and versatile.

## Graphical abstract

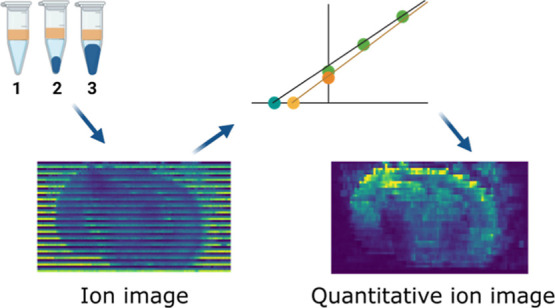

Mass spectrometry imaging (MSI) is a rapidly growing technique for chemical characterization of biological samples.^1^ With MSI, it is possible to obtain spatial distributions of ionizable endogenous molecules, drugs, and drug metabolites in a single experiment without preselection.^1,2^ In a typical MSI experiment, mass spectra are acquired directly from a thin tissue section and contain hundreds of analytes at each location. Subsequently, ion images of individual analytes are generated in two dimensions by mapping ion intensities as a function of location on the sample.^2,3^ However, signal intensities in mass spectra can be affected by factors other than analyte concentrations, which makes quantification in MSI experiments challenging.^3–6^ Factors that may distort ion images include matrix effects, particularly ion suppression effects, and adduct formations, which occur during the ionization of analytes as molecules compete for charge.^3,7–9^ Furthermore, while several approaches have been proposed to estimate local extraction efficiencies,^10,11^ these values are often unknown in MSI; therefore, detected concentrations are typically reported instead. Although matrix effects are present in any mass spectrometry-based method, they are of particular concern in MSI when molecules from a specific location on the sample surface are ionized simultaneously. Therefore, to achieve accurate quantification, it is imperative to compensate for matrix effects in quantitative MSI (Q-MSI).

The most commonly employed procedure to compensate for matrix effects is to add carefully selected, and often isotopically labeled, internal standards (IS) that can be used for normalization and quantitation.^6,12^ Strategies to include IS vary between MSI techniques and include spotting the standard on top of the tissue section and doping the extraction solvent.^6^ Doping the extraction solvent with IS is particularly useful in ambient ionization liquid extraction-based MSI techniques.^13^ In this approach, the IS are present at a constant concentration throughout the entire imaging experiment. Thus, the analyte and its corresponding IS are ionized and detected simultaneously, which enables pixel-to-pixel quantification using one-point calibration.^14–16^ However, the use of isotopically labeled IS is not without drawbacks, as these compounds can be expensive or commercially unavailable. An alternative to quantitation with IS (qIS) in chemically complex matrices is quantitation using standard addition (qSA). The qSA approach is commonly used in analytical chemistry and relies on adding known quantities of a nonlabeled analyte to the sample. Next, the measured increase in signal is correlated to the added concentration.^17^ Provided that the responses from the unknown sample and additions are linear, it is possible to quantify analytes by extrapolation to zero response.^18,19^ The qSA approach is especially appropriate when the sample composition is unknown or complex and is anticipated to affect the analytical signal. The advantage of qSA is that it accounts for the rotational matrix effects, i.e., effects changing the slope of a calibration curve.^18^

The qSA approach has been widely used in spectrophotometric analysis^20–22^ but has only gained comparatively minor recognition in mass spectrometry-based techniques. This may be related to the commonly cited disadvantage of increased sample consumption and a lengthy sample preparation step for qSA compared to qIS. Nevertheless, qSA has been adopted, for instance, in liquid chromatography–mass spectrometry to compensate for matrix effects in vitamin D assay,^23^ in liquid chromatography–tandem mass spectrometry for quantification of gluten-derived metabolites in urine samples,^24^ and for the quantification of metformin in post-mortem blood.^25^ In gas chromatography–tandem mass spectrometry, qSA was, for example, employed to overcome matrix effects when analyzing pesticides in food commodities^26^ and in high-throughput direct infusion nanoelectrospray mass spectrometry for parallel targeted analysis of multiple metabolites in urine samples.^27^ In MALDI MSI, standards have been spotted on tissue to use a qSA approach in combination with IS for quantification of acetyl-l-carnitine and cocaine in piglet brain tissue.^28,29^ However, the approach still required isotopically labeled standards, and quantitative ion images could not be obtained. Thus, a Q-MSI approach using qSA, where quantitative ion images are obtained, has not been previously accomplished.

Here, we demonstrate the first approach for generating quantitative ion images obtained with qSA. These were acquired by doping the solvent of pneumatically assisted nanospray desorption electrospray ionization (PA nano-DESI) with increasing standard concentrations in alternating line scans. We validated the qSA approach by comparing it to both qIS and quantification with an external calibration curve (qEC) and report similar results for qSA and qIS. Furthermore, we show that it is possible to push the number of analytes simultaneously quantified by qSA using endogenous molecules extracted from tissue as a standard mixture. In addition to increasing the number of analytes that can be quantified, the use of extracted endogenous molecules overcomes the limitation of standard availability. Overall, we find that our qSA approach will be highly valuable for Q-MSI of endogenous species, in particular, when isotopically labeled standards are not available.

## Materials and Methods

### Tissue Collection and Handling

Fresh-frozen mouse brains were purchased from Creative Biolabs (NY, USA) and sectioned using a cryotome (Leica CM1900, Leica Microsystems, Wetzlar, Germany) at 12 μm thickness. The sections were thaw-mounted on regular microscope glass slides (Fisher Scientific, Gothenburg, Sweden) and stored at −80 °C before analysis.

### Reagents and Materials

Methanol (≥99.8%) was purchased from VWR International (Radnor, PA, USA). γ-aminobutyric acid (GABA, ≥99%) and ^15^N-labeled amino acid mix (≥99%) containing l-alanine, l-arginine hydrochloride, l-asparagine, l-aspartic acid, l-cysteine, l-glutamic acid, l-glutamine, Glycine, l-histidine hydrochloride, l-leucine, l-isoleucine, l-lysine hydrochloride, l-methionine, l-phenylalanine, l-proline, l-serine, l-threonine, l-tryptophan, l-tyrosine, l-valine, and formic acid (FA) (98–100%) were purchased from Sigma-Aldrich (Darmstadt, Germany). d­(+)-Glucose standard was bought from BDH Laboratory Supplies (Poole, UK), and chloroform (99+%) was from Thermo Fisher Scientific (Waltham, MA, USA). Water (resistivity of 18.2 Ω) was acquired from a Milli-Q laboratory water purifier (Millipore, Bedford, MA, USA).

Rat brain extract (RBE) was prepared as follows: 1.17607 g of rat brain was placed in 33.976 mL of methanol. Afterward, the rat brain was homogenized using an ultrasonic bath and centrifuged. The supernatant was collected and centrifuged once again. The final concentration of RBE stock solution was 34.6 mg mL^–1^ of wet rat brain. The extract was further diluted using methanol/mQ water v/v 9:1 + 0.1% FA to the desired concentrations.

For selected experiments, the RBE was cleaned up using liquid–liquid extraction (LLE), where 500 μL of 34.6 mg mL^–1^ RBE was pipetted in a 20 mL glass vial. The solution was mixed with 500 μL of chloroform and 700 μL of mQ water, vortexed, and shaken vigorously for 30 s. The vial was placed into a microcentrifuge (MiniStar Silverline, VWE International, Radnor, PA, USA) for 2 min to speed up the phase separation. The aqueous-rich phase was collected, transferred into Eppendorf tubes, and vacuum-dried using a centrifuge concentrator (Concentrator Plus, Eppendorf, Hamburg, Germany). Subsequently, the sample was reconstituted in 1 mL of methanol/mQ water v/v 9:1 + 0.1% FA and further diluted to the desired concentrations.

### Sampling and Imaging with PA Nano-DESI

The PA nano-DESI probe was constructed based on the design of Duncan et al.^30^ Briefly, two fused silica capillaries (50 × 150 μm, i.d. × o.d., Polymicro Technologies, L.L.C., Phoenix, USA) were positioned in a 3D-printed cassette at a fixed angle (∼90°) and nitrogen gas was supplied at ∼5 bar.^31^ The solvent was propelled using a syringe pump (Legato 180, KD Scientific, Holliston, USA) at a flow rate of 0.5 μL min^–1^. The PA nano-DESI solvent consisted of methanol/mQ water v/v 9:1 + 0.1% FA, spiked with increasing concentrations of either (i) amino acid standards (AA), (ii) RBE subjected to liquid–liquid extraction to remove signal-suppressing lipids (LLE-RBE), or (iii) untreated RBE. Additionally, a cocktail of IS was spiked into the solvent; concentrations of standards and dilutions of RBE in different experiments can be found in Tables S1 and S2, respectively. Experiments were conducted in both touchdown mode and MSI mode.

Touchdown experiments were recorded in the prefrontal cortex of brain tissue by simply parking the PA nano-DESI probe on the selected spot and keeping it there for >20 s. The probe remained on each spot until the intensity of a base peak characteristic for the tissue (creatine at *m*/*z* 132.0756) decreased in intensity to 1% in relative abundance, assuming exhaustive extraction from that spot. Triplicate data were acquired for each solvent composition. In touchdowns using LLE-RBE, the signal of PC 34:1 at *m*/*z* 760.5856 was monitored as it was not present in the extraction solvent. For solvents containing RBE, the creatine signal was monitored, and the probe stayed on the spot until the signal reached the relative abundance of the baseline level.

In imaging experiments, the sample was moved at a speed of 0.04 mm s^–1^ under the PA nano-DESI probe along the *x*-axis, and each acquired line with the same solvent composition was spaced by 450 μm along the *y*-axis. Lines containing other solvents were acquired with a 150 μm offset from the previous. The motion and distance between the sample and the probe were controlled by a motorized linear stage with *XYZ* configuration (Zaber Technologies Inc., Vancouver, BC) operated via a LabVIEW program, which accounted for the tilt of the glass slide where the sample resided.^32^ Considering the scan rate of the mass spectrometer (1.96 scans s^–1^), the final pixel size was ∼20 × 150 μm^2^.

For qSA imaging, three different solvents with three different standard concentrations were used as the nano-DESI solvent. The ion image was generated by first recording data using only solvent 1, leaving sufficient space to later record data using solvents 2 and 3 between the line scans of solvent 1.

### Mass Spectrometry

All data were recorded using a QExactive mass spectrometer (Thermo Fisher Scientific, Bremen, Germany) operated in positive mode at a resolution of 140,000 (*m*/Δ*m* at *m*/*z* 200), Orbitrap AGC target of 1 × 10^6^, S-lens RF level of 60, heated ion transfer capillary temperature of 275 °C, and electrospray voltage at 3.8 kV. Maximum injection time was set to 300 ms, although the actual injection time was kept below 10 ms. MS spectra were acquired in the 70–300 *m*/*z* mass range of amino acid additions and in the 70–1000 *m*/*z* mass range for RBE additions.

### Data Processing

All data files were converted from RAW files (XCalibur, Thermo Fisher Scientific) to centroided mzML files using ProteoWizard MSConvert version 3.0.^33^ For touchdown experiments, the raw files were sliced using XCalibur RawFile Slicer to only contain 12 s of data, during which time exhaustive extraction of AAs was achieved. Next, using a custom MATLAB script (MATLAB R2022b, MathWorks, USA), the detected features were aligned across all files (5 ppm mass tolerance) using the alignment function developed for i2i.^34^ The signal intensities were normalized to the total ion count (TIC) and integrated for each extraction peak (Figure S1). For processing of MSI data, mzML files containing line scan data were input into an in-house MATLAB-based i2i software,^34^ where detected features were aligned (5 ppm mass tolerance) and normalized to TIC in each pixel to obtain “raw” ion images (a matrix of intensities). The resulting matrix was converted into quantitative ion images using a custom script (https://github.com/LanekoffLab/). Calculations, including least-squares regressions, the statistical significance of mean concentrations between quantification methods, and regions of interest (ROI) quantitative analysis were performed in RStudio. Prior to statistical analyses, the normality of data was tested, and in case normality could not be assumed, nonparametric test options were chosen. The type of statistical test is specified, along with each comparison. For standard deviation calculations, triplicate measurements were used for internal standard quantification (qIS) and external calibration quantification (qEC), and eq S1, including the uncertainty of the slope and the intercept, was used for standard deviation calculation for quantification by means of standard addition (qSA).^35^

## Results and Discussion

### Comparison of Three Quantification Approaches

The three main ways to quantify analytes in analytical chemistry are external calibration (qEC), one-point calibration with an internal standard (qIS), and standard addition (qSA). In Q-MSI, quantification of endogenous analytes in tissue using qIS is considered the gold standard. By using labeled IS in the extraction solvent, qIS is easily accomplished in nano-DESI MSI by calculating the detected concentrations pixel-by-pixel.^36,37^ Despite having a similar analyte extraction efficiency in various regions, the absolute amounts are expected to vary between regions.^36,38^ Contrarily to qIS, qEC relies on establishing a calibration curve of nonlabeled standards prior to MSI for calculating the concentration in each pixel using the regression curve.^39^ The same nonlabeled standards can be used for qSA, which is based on adding multiple standard concentrations to the sample, measuring the signal increase, and extrapolating to zero response. Although qSA has not been previously described for Q-MSI, it holds promise for providing detected concentrations of endogenous analytes while accounting for matrix effects across different tissue regions without the need for isotopically labeled standards.

One molecular class of interest in biological tissues is amino acids (AA), which play important roles in neurological health and function. Altered AA concentrations are associated with numerous neurological processes and disease states.^40,41^ In an experiment designed to quantify endogenous AA in mouse brain tissue, PA nano-DESI was employed to simultaneously perform qEC, qIS, and qSA. The experimental design included six different standard compositions. All different solvents that included standards were used as the extraction solvent, i.e., the standards were incorporated directly in the nano-DESI solvent, and therefore, all standards were always present during ionization. Solvent 1 only included ^15^N-labeled IS for qIS. In addition to IS, solvents 2–6 also included 20 nonlabeled AA standards at increasing concentrations for qSA and qEC (Figure 1, Table S1). The selected concentrations for qEC and qSA were based on preliminary results, while the individual concentrations of AA for qIS were determined by the precombined standard mixture. All quantitative data were acquired with PA nano-DESI in touchdown mode. In short, the probe was positioned in contact with selected locations on the prefrontal cortex of mouse brain tissue, where it stayed until the analyte signals decayed (Figure 1). Data for qEC were acquired before each touchdown; data for qIS were acquired from touchdowns with solvent 1, and data for qSA were acquired from touchdowns with solvents 1–6. The subsequent touchdowns were closely spaced (∼200 μm) to minimize variations due to natural analyte gradients in tissue (Figure S2). Thus, the experiment was designed to keep the overall variations minimal.

**Figure 1:**
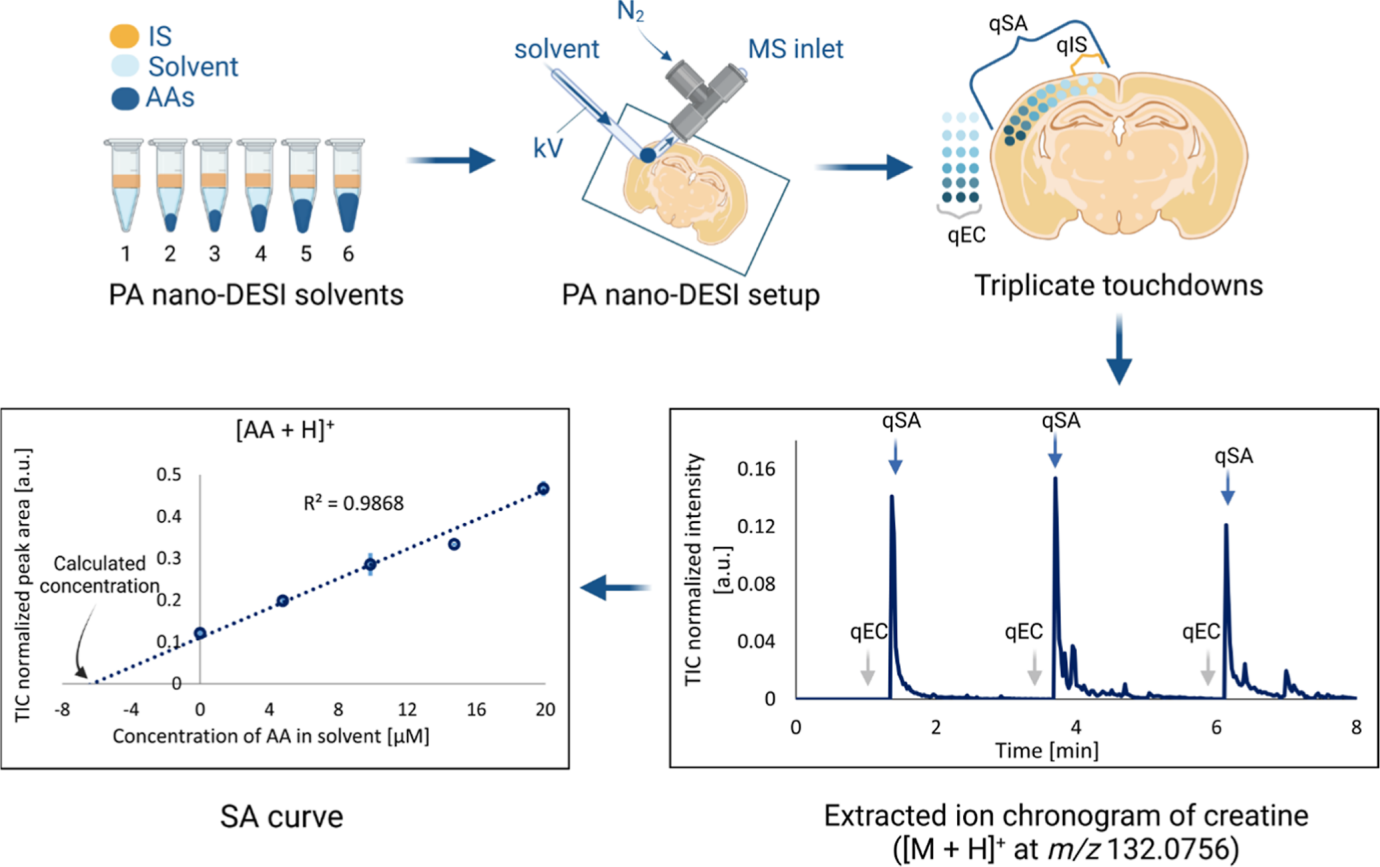
Experimental workflow for quantifying AA in mouse brain tissue with three different quantitative approaches. Top row: six standard compositions were prepared in solvent (light blue) with constant concentrations of IS (yellow) and increasing concentrations of AA standards (dark blue). Each solvent was used when the PA nano-DESI probe sampled endogenous AA from selected spots in the cerebral cortex, with triplicate touchdowns for each solvent composition. Bottom row: extracted ion chronogram of creatine (*m*/*z* 132.0756) used to identify time-points of the touchdown for data extraction of all AA for qEC, qSA, and qIS, with the concentration for qSA calculated using extrapolation.

The results show that 19 out of 20 endogenous AAs were detected from tissue, in at least one adduct form, for quantification based on the touchdown experiment. Cys was not detected in any adduct form, presumably owing to its poor ionizability in positive ionization mode.^42^ Quantification for qIS used eq S2, and six-point qSA and qEC regression curves were constructed using summed TIC normalized intensities (Figures S3 and S4). For all AAs, the adduct with the highest signal intensity was used, which in most cases was the protonated adduct ([M + H]^+^) except for Ser and Asn, where the sodiated adduct ([M + Na]^+^) was more abundant. Despite differences in abundances and physicochemical properties of AAs, the regression curves for qSA showed good linearity (ANOVA on residuals, α = 0.05) and an average coefficient of determination *R* ^2^ > 0.8 (Figures 2A–D and S3). The regression curves for qEC also showed high linearity, while the slopes for qEC were steeper than those for qSA (Figure S4).

**Figure 2:**
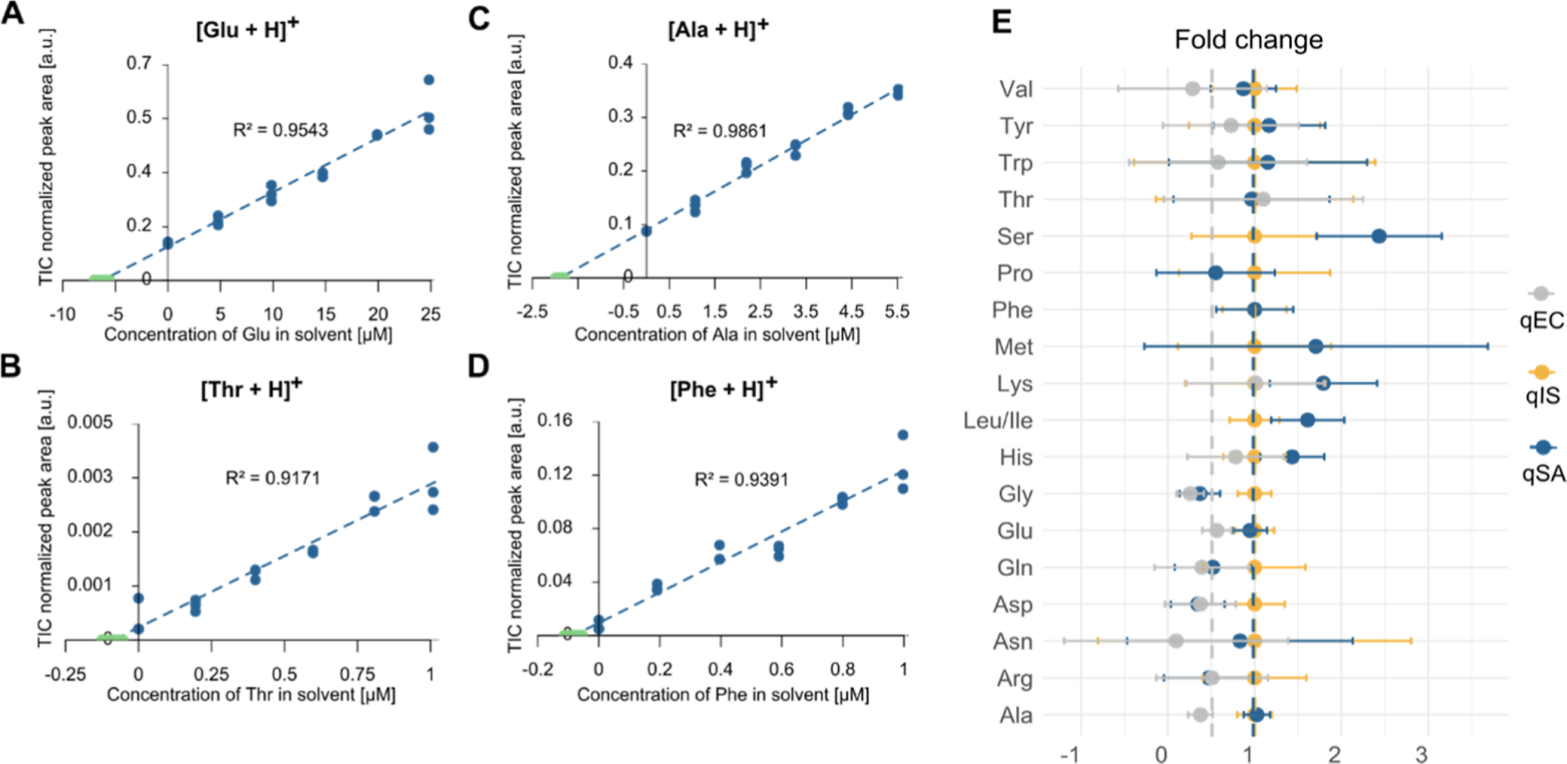
Comparison of three quantitative approaches for quantifying AA in tissue using touchdown extractions. Curves for qSA of (A) Glu, (B) Thr, (C) Ala, and (D) Phe with the standard deviation (*n* = 18) of the calculated concentration marked in green on the *x*-axis, *n* = 3 for each concentration. (E) Comparison of detected concentrations of endogenous amino acids calculated using qIS, qSA, and qEC. Values in (E) are plotted relative to the detected concentrations obtained by qIS. Error bars for qIS, qSA, and qEC correspond to the combined standard deviations from triplicate measurements for qIS, ±uncertainty for qSA, and triplicates for qEC. Colored dotted lines correspond to the median fold change of the respective quantification approach, qEC (gray), qIS (yellow), and qSA (blue).

The performance of qSA and qEC was compared to the commonly used qIS across multiple amino acids based on the touchdown data. When comparing the quantitative results from qSA and qIS, no significant differences were found (paired two-tailed *t*-test, α = 0.05) (Figures 2E and S5, Table S1). Note that the relative standard deviations were 37% for qSA and 47% for qIS, showing a similar confidence interval between the two approaches. Overall, this suggests that qSA is similar to qIS since matrix effects are effectively accounted for and that the quantitative variability in detected endogenous AA concentrations is well described (Table S1).^43^ When comparing the results of qEC- and qIS-derived concentrations, qEC significantly underestimates the endogenous concentrations (paired two-tailed *t-*test, α = 0.05). This indicates that qEC cannot account for the ion suppression to the same extent as qIS. For example, it was not possible to quantify Leu/Ile, Met, Phe, Pro, or Ser with qEC since the calculated concentrations became negative. Furthermore, relative to qIS, the median fold change for qEC is only 0.51, while for qSA it is more similar to qIS with 0.98 (Figure 2E). In summary, the similarity between qSA and gold standard qIS shows that qSA is a viable alternative to qIS for quantifying endogenous metabolites in Q-MSI.

### Quantification with qSA for Q-MSI

Generating quantitative ion images with qSA requires tissue sampling using different standard concentrations. Therefore, we established a workflow using alternating line scans with three different extraction solvents: solvent 1 contained IS; solvent 2 contained a low concentration of AA standards; and solvent 3 contained a high addition of AA standards (Figure 3A, Table S1). First, all line scans containing solvent 1 were recorded and spaced by 450 μm to leave room for line scans using solvents 2 and 3, resulting in a final spacing of 150 μm between line scans (Figure 3B,C, Supporting Information 2). The recorded line scans were visualized using our i2i software,^34^ producing ion images with “stripes”, characteristic for the increasing AA concentrations (Figure 3D). These raw ion images were converted into quantitative ion images using a newly developed in-house MATLAB script. Specifically, a block average of 8 TIC normalized pixels was added as the first calibration point in an SA regression curve (solvent 1). Following, 8-pixel block averages of the two subsequent line scans (solvent 2 and 3) were added and together they formed the regression curve for qSA (Figure 3E). Finally, the concentration value was obtained by extrapolation to a zero response and saved as one pixel in the quantitative ion image. By moving the reading frame one step in either *X* (8 × 20 μm = 160 μm) or *Y* (150 μm) direction, the concentration was calculated in all pixels of the ion image (Figures 3F and S6), while keeping the final spatial resolution at 160 × 150 μm (*X* × *Y*). The number of pixels used for the block average was selected to gain the final squared pixels, excluding any measurement violating linear regression. This workflow, which is based on three line scans with different standard concentrations, enabled the first construction of quantitative ion images with qSA.

**Figure 3:**
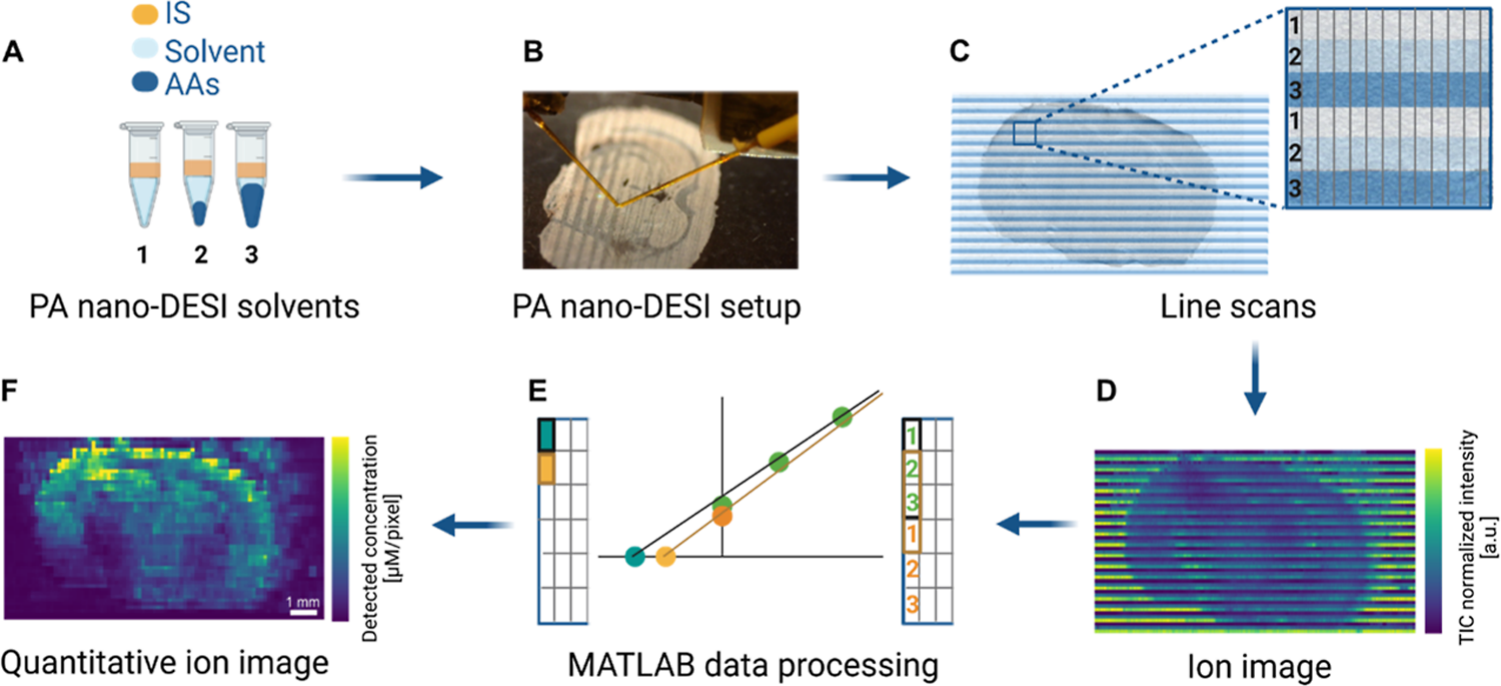
Workflow for Q-MSI using qSA. (A) Three solvent compositions were prepared with constant concentrations of IS and increasing concentrations of AA standards. (B) The PA nano-DESI MSI setup was applied to acquire line scans of the whole mouse brain tissue section. (C) Line scans were acquired with PA nano-DESI MSI using three alternating solvent compositions. (D) Ion image created using i2i software.^34^ (E) Data points from three adjacent pixels were used to calculate the *x*-intercept, the detected concentration, using a MATLAB data processing script. (F) Quantitative ion image based on qSA generated using the MATLAB script. The scale bar in the bottom right corner corresponds to 1 mm.

The quantitative ion images based on qSA show a large span in AA concentrations and dynamics over the tissue (Figures 4A–D and S7). For example, detected concentrations of Glu go up to 40 μM while Phe is only detected at submicromolar concentrations (Figure 4A–D). Being a major excitatory neurotransmitter, the detected concentration of Glu is highest in the cortex, hippocampus, and striatal regions. This is in good agreement with previous studies^44,45^ and with the reported distribution of Glu transporters.^46,47^ Neurotransmitter precursors and modulators, such as Ala, Phe, and Thr, show the highest detected concentrations in the hippocampal and cortex regions with high levels of Thr in the hypothalamus, which matches well with previous reports.^48^ The spatial distributions of various amino acids in brain tissue regions show that all detected amino acid concentrations are higher in gray matter compared to white matter (Figure 4A–D). Thus, the qSA-derived quantitative ion images produce reliable information on chemical distributions in tissue.

**Figure 4:**
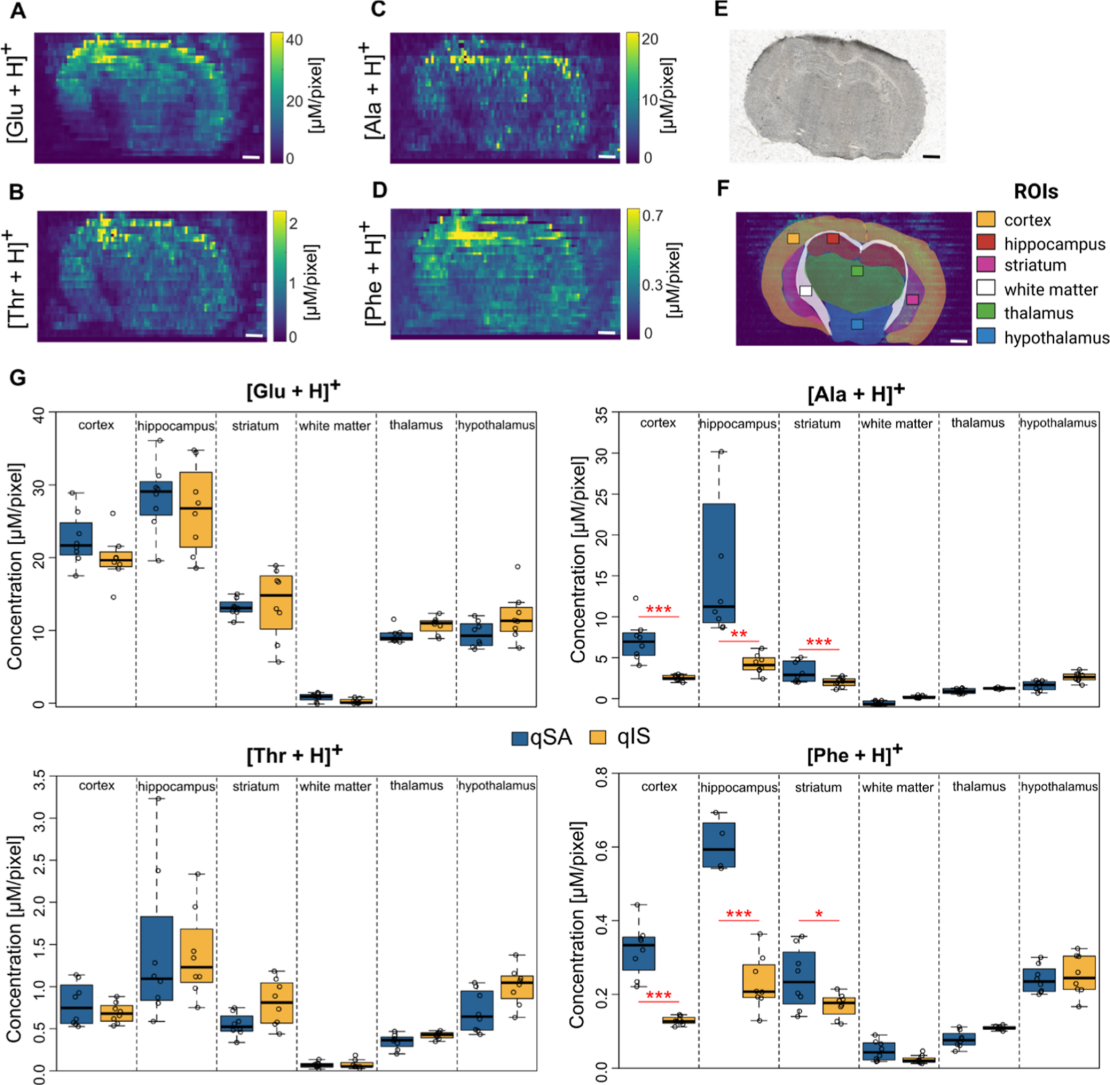
Quantitative ion image based on qSA and quantitative ROI analysis. (A–D) Quantitative ion images derived from qSA regression for four selected amino acids: (A) Glu, (B) Thr, (C) Ala, and (D) Phe (more in Figure S7), filtered to the 99th percentile of concentrations. (E) Optical image of a mouse brain tissue section used. (F) Map of the selected ROIs. (G) Comparison of qSA (blue) and qIS (yellow) in 6 mouse brain regions for four amino acids (more in Figure S9); the *y*-axis shows the detected concentration in μM pixel^–1^. The scale bar corresponds to 1 mm.

The quantitative ion images obtained with qSA were further compared to those obtained with qIS in selected ROIs. Specifically, six different ROIs were defined on the mouse brain tissue based on a visual comparison of the optical image (Figure 4E) with the Allen Brain Atlas.^49^ Within each brain region, an ROI corresponding to an area of 640 × 600 μm (*x* × *y*) was selected (Figure 4F). All defined ROIs included four lines, two with solvent 1, one with solvent 2, and one with solvent 3 (Figure S8). When comparing the qIS- and qSA-derived ROI concentrations for Glu, Thr, Ser, and Asn, no significant differences were detected (aligned rank transform, α = 0.05) (Figures 4G and S9). However, a difference in certain regions was found for other amino acids (including Ala and Phe), in particular, in the cerebral cortex, hippocampus, and striatum (Figures 4G and S9). Plausible explanations include the use of nonideal standard concentrations, mainly due to restrictions in premixed standard mixtures. Furthermore, the dynamics of natural AA concentration gradients in tissue may contribute since the ROIs were quite large, covering 600 μm^2^. Thus, for future applications of qSA, it is advisable to use the smaller step sizes achievable with a smaller nano-DESI probe to gain smaller pixel sizes.^50–53^ Nevertheless, our proof-of-principle data clearly shows that qSA can be used in Q-MSI without the need for isotopically labeled standards.

### Nontargeted qSA for Q-MSI

Without the need for labeled standards, the qSA approach has the potential to be expanded into a nontargeted approach. Here, we move toward a fully nontargeted approach by using dilutions of endogenous molecules extracted from rat brain tissue as a source of calibration standards for qSA. In addition to being readily available, the extract includes virtually all analytes of interest when imaging a mouse brain tissue section, thereby providing the unique opportunity to quantify numerous analytes simultaneously within a single MSI experiment without preselection. Two types of methanolic RBEs were compared: a nontreated extract (RBE) and an extract cleaned up to remove potentially signal-suppressing phospholipids (LLE-RBE). When comparing the mass spectra, the RBE showed similar chemical complexity as the sampled cortex of a tissue section, with more than 1800 detected features, while the complexity of the LLE-RBE was reduced (Figure S10). However, despite the reduced chemical complexity, the LLE-RBE contained more than 600 *m*/*z* features that could be used for qSA. For qSA of amino acids of both touchdown and MSI data, we found that using the LLE-RBE provided improved linearity, reduced standard deviations, and enhanced image quality compared to RBE (Figure S11). Additionally, LLE-RBE provided quantities similar to those of qIS (Figure S12). Overall, the LLE-RBE was found superior for nontargeted qSA of small polar metabolites.

One class of small polar metabolites of importance is neurotransmitters, which mediate signaling between neuronal cells in the brain.^48,54^ Fluctuations in their concentrations are associated with numerous neuronal functions and dysfunctions, and pathological changes in neurodegenerative diseases such as Parkinson’s disease, Alzheimer’s disease, and multiple sclerosis.^55,56^ Thus, gaining accurate information about their amounts and distributions is crucial for understanding their role in health and disease. In an experiment, LLE-RBE was spiked into the extraction solvent at increasing concentrations, and a mouse brain tissue section was imaged using PA nano-DESI using the established qSA workflow. The unprocessed ion images of five selected neurotransmitters show clear stripes of increasing intensity related to the increased amount of LLE-RBE in the nano-DESI solvent (Figure 5A). However, when the data are run through our data processing workflow, the stripes disappear, the pixels enlarge, and the ion images become quantitative with the color scales depicting the detected concentrations (Figure 5B). Note that the detected concentrations are expressed relative to the LLE-RBE amount in mg/mL of LLE-RBE/pixel, which can be converted to μM/pixel when the analyte concentrations in the LLE-RBE are known (Figures 5C and S13). Thus, the nontargeted qSA can provide unique insights into concentration differences between tissue regions.

**Figure 5:**
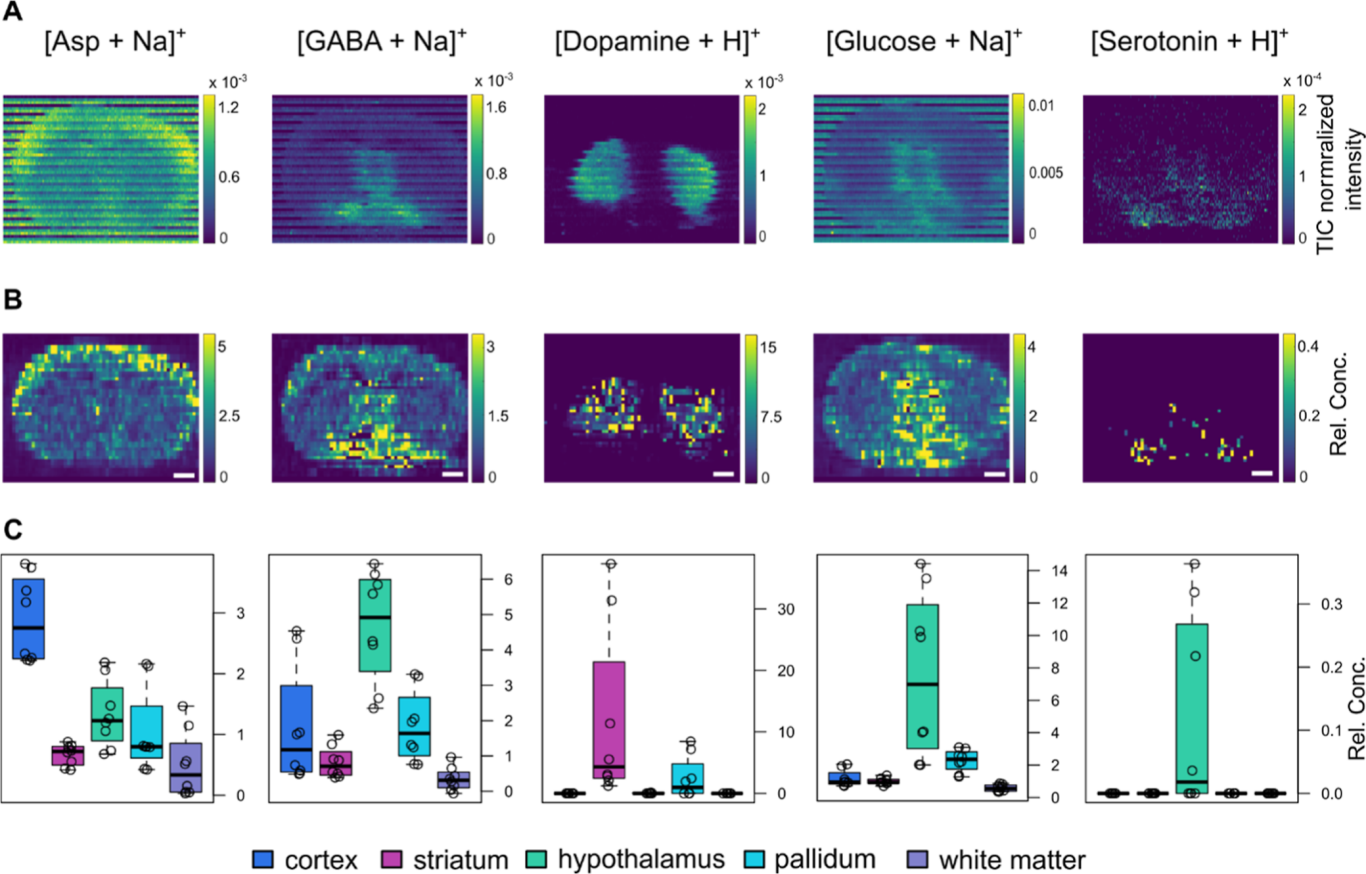
Nontargeted Q-MSI using qSA displaying neurotransmitters in mouse brain tissue. (A) Raw images visualized in i2i software,^34^ scale bar shows TIC normalized intensities. (B) Quantitative ion images generated with SA; scale bar depicting relative concentration. Images are filtered to the 99th percentile of concentrations. (C) Plots showing relative concentration in five different ROIs of the brain tissue, left to right: cortex (blue), striatum (magenta), hypothalamus (green), pallidum (light blue), and corpus callosum (purple), with each circle representing one pixel in the respective ROI. For each analyte, the most abundant adduct was used. Relative concentrations (Rel. Conc.) are relative to the endogenous amount of the respective analyte present in LLE-RBE (mg tissue/mL solvent).

The spatial distribution of neurotransmitters was evaluated by defining 5 ROIs on the tissue (Figure S14), which reveals distinct localization patterns. Asp exhibited elevated levels in the cortex, consistent with previous studies.^57^ Furthermore, γ-aminobutyric acid (GABA) was predominantly localized in the hypothalamus. This aligns with earlier reports on its high prevalence in the medial septum/diagonal band region, which provides significant GABAergic input to the hippocampus.^57,58^ Dopamine was primarily concentrated in the striatum, in agreement with previous reports,^44,46,59,60^ and serotonin showed a similar spatial distribution as in previous MSI experiments of similar coronal mouse brain sections.^61,62^ Glucose was detected in all selected ROIs; however, the highest abundance was recorded in the hypothalamus and pallidum. These findings demonstrate the versatility of our quantitative imaging approach in accurately mapping the distribution of neurotransmitters across brain regions, providing valuable insights into neurochemical variations relevant to neurological research.

In addition to neurotransmitters, a wide range of metabolites can be detected and quantified to follow the metabolic pathways of interest (Figure S15). Furthermore, the qSA approach significantly enhances the sensitivity for low-abundant analytes that would otherwise remain undetectable without the addition of standards (Figure S16). Overall, despite the increased chemical complexity introduced by the endogenous standard mixture, the results demonstrate that the qSA concept was successful. Thus, we present not only a novel quantitative approach but also a transformative strategy for conducting nontargeted Q-MSI. The increased flexibility of nontargeted Q-MSI allows for expanding the use of the data beyond initial hypotheses and targeted analytes.

## Conclusion

In this work, we present the first combination of qSA and MSI by employing PA nano-DESI Q-MSI. We show that qSA provides quantities similar to those of qIS and that qEC significantly underestimates the concentrations since it does not account for matrix effects. In addition to our approach circumventing the need for isotopically labeled IS, we show that analytical standards can be exchanged for endogenous analytes extracted from biological material, such as tissue or cells, without losing quantification accuracy. Although our study uses a moderate spatial resolution, this can be improved in future studies. Importantly, the elimination of standard preselection allows for nontargeted relative quantification in MSI at any spatial resolution. We find that our qSA workflow is cost-effective, accessible, and versatile, and we anticipate that this approach will provide a foundation for future nontargeted quantification in both MS^1^I and MS^2^I.

## Supplementary Material





## Notes

Equations, camera images, calibration curves, graphs, ion images, and tables (PDF)
Video showing PA nano-DESI MSI sampling (AVI)
