# Supplementary material for: Standard Addition as a Method for Quantitative Mass Spectrometry Imaging
Source: Anal Chem. 2025 May 27;97(22):11572–80. doi: 10.1021/acs.analchem.5c00549 (PMC12163875; doi:10.1021/acs.analchem.5c00549)
Supplement: Supplementary file 1 [file ac5c00549_si_001.pdf]

Supporting Information

Standard addition as a method for quantitative mass spectrometry imaging

Lucie Davidová[1] and Ingela Lanekoff[1,2*]

[1]Department of Chemistry – BMC, Uppsala University, Box 576, 751 23 Uppsala, Sweden

[2]Center of Excellence for the Chemical Mechanisms of Life, Uppsala University, 751 23 Uppsala, Sweden

*Corresponding author

Prof. Ingela Lanekoff,

Department of Chemistry – BMC,

Husargatan 3, 75 123 UPPSALA, Sweden.

Email: ingela.lanekoff@kemi.uu.se

Supporting Information Content

Contents

Equations S3

Figures S4

Tables S19

References S20

Equations

Equation S1

Equation for calculating standard deviations for qSA:[1]

As both the intercept and slope of the SA curve are subject to error, the formula for the standard deviation, s_{xE}, of the extrapolated x-value (x_E) is calculated using:

$$s_{x_E} = \frac{s_{y/x}}{b}\sqrt{\frac{1}{n} + \frac{\bar{y}^2}{b^2 \sum_i (x_i - \bar{x})^2}},$$

Where b is the slope of the curve, n is the number of data points and $s_{y/x}$ are random errors in the y-direction, calculated as:

$$s_{y/x} = \sqrt{\frac{\sum_i (y_i - \hat{y}_i)^2}{n-2}},$$

Where $\hat{y}_i$ are the fitted y-values.

Equation S2

Equation for one-point qIS:[2]

The detected concentration ($c_{detected}$) in the PA nano-DESI solvent can be calculated using qIS as:

$$c_{detected} = \frac{I_{end}}{I_{IS}} \cdot c_{IS},$$

Where I_{end} is the signal intensity of endogenous analyte, I_{IS} is the signal intensity of the corresponding internal standard and c_{IS} is the corresponding internal standard concentration.

Figures

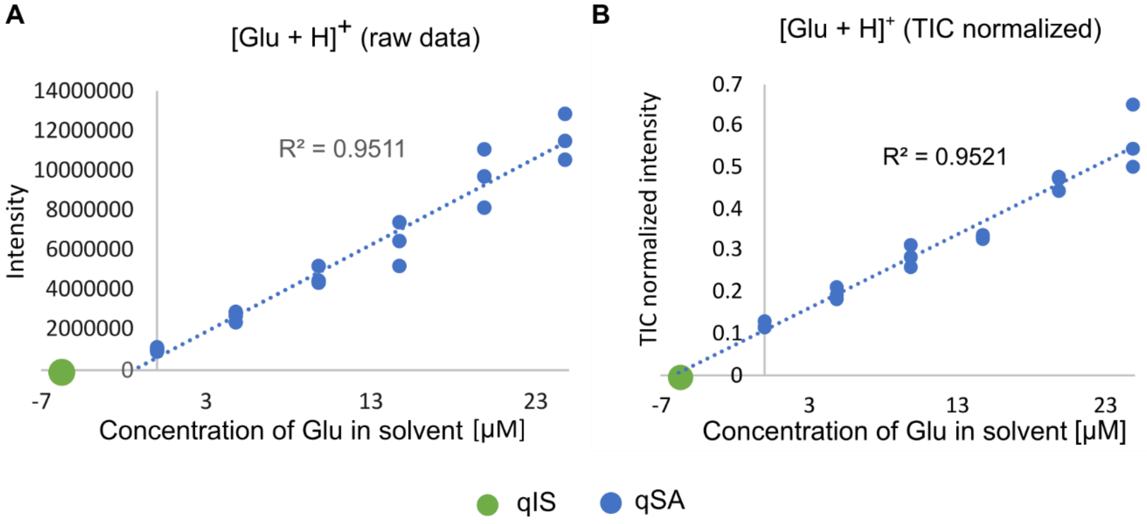

Figure S1. Comparison of data treatment strategies for qSA using data from a touchdown experiment with Glu standard spiked into extraction solvents (1-6). (A) Regression curve of non-normalized qSA data (blue). (B) Regression curve of TIC normalized qSA data (blue). Normalization with TIC prior to qSA generates less variation within each calibration point, a slightly higher coefficient of determination (R^2), and a closer match to qIS-derived concentration (green).

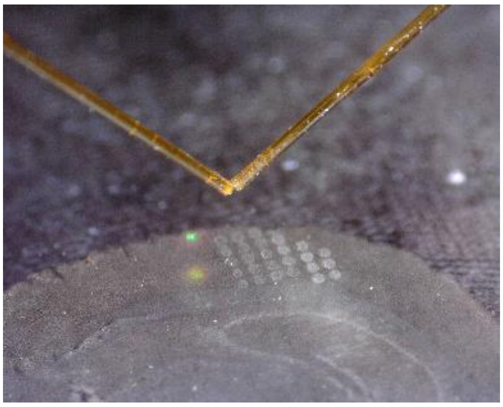

Figure S2. Picture showing spots after PA nano-DESI touchdowns in the prefrontal cortex of a mouse brain tissue. The sampled spots are distanced by 200 µm, ensuring no overlap in desorbed areas, while inferring homogeneous endogenous analyte concentrations.

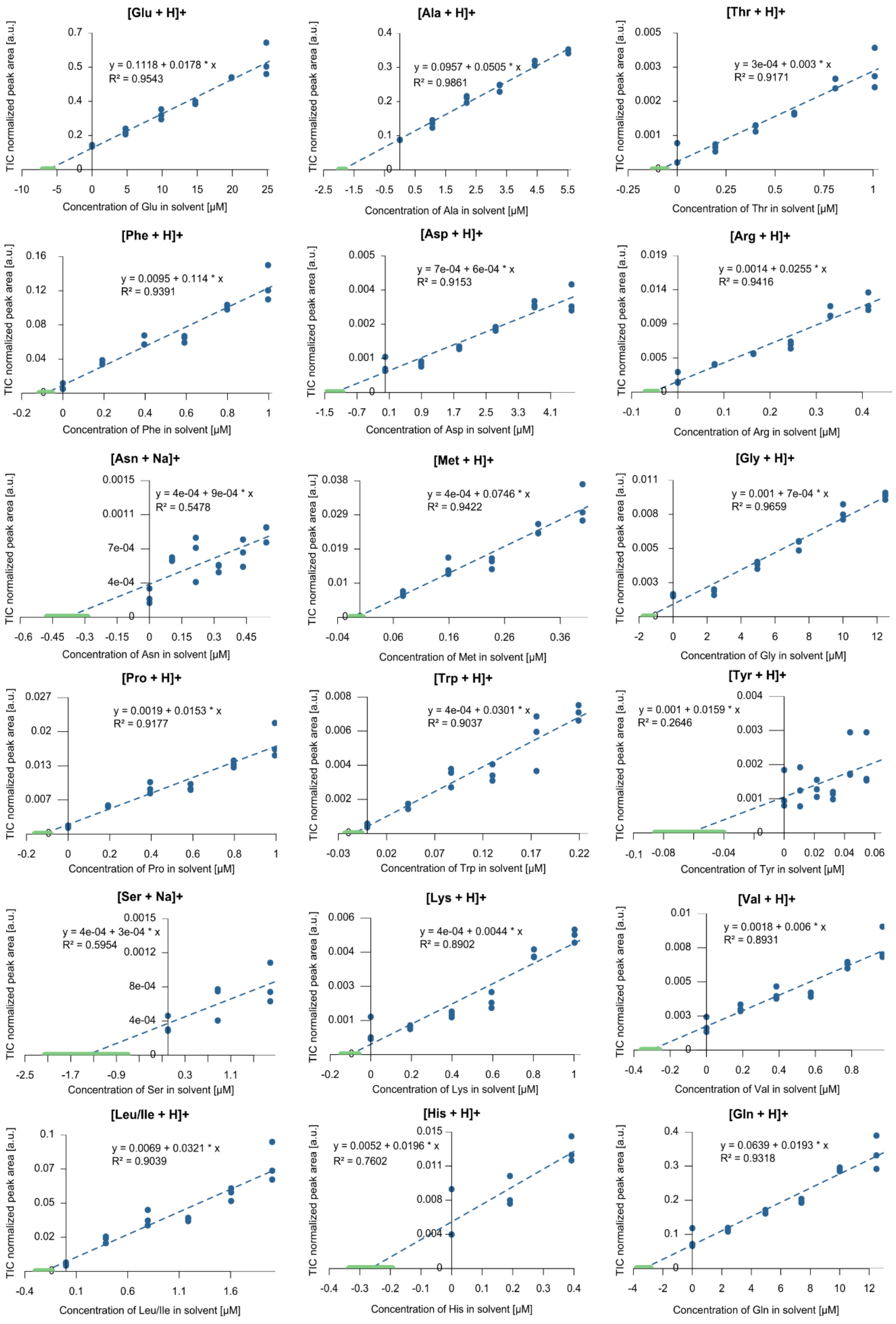

Figure S3. qSA regression curves for amino acids using standards. For amino acid standard response that was not linear over the entire range of concentrations, only the null addition and the two lowest concentrations are plotted. The extrapolated concentration interval is visualized in green.

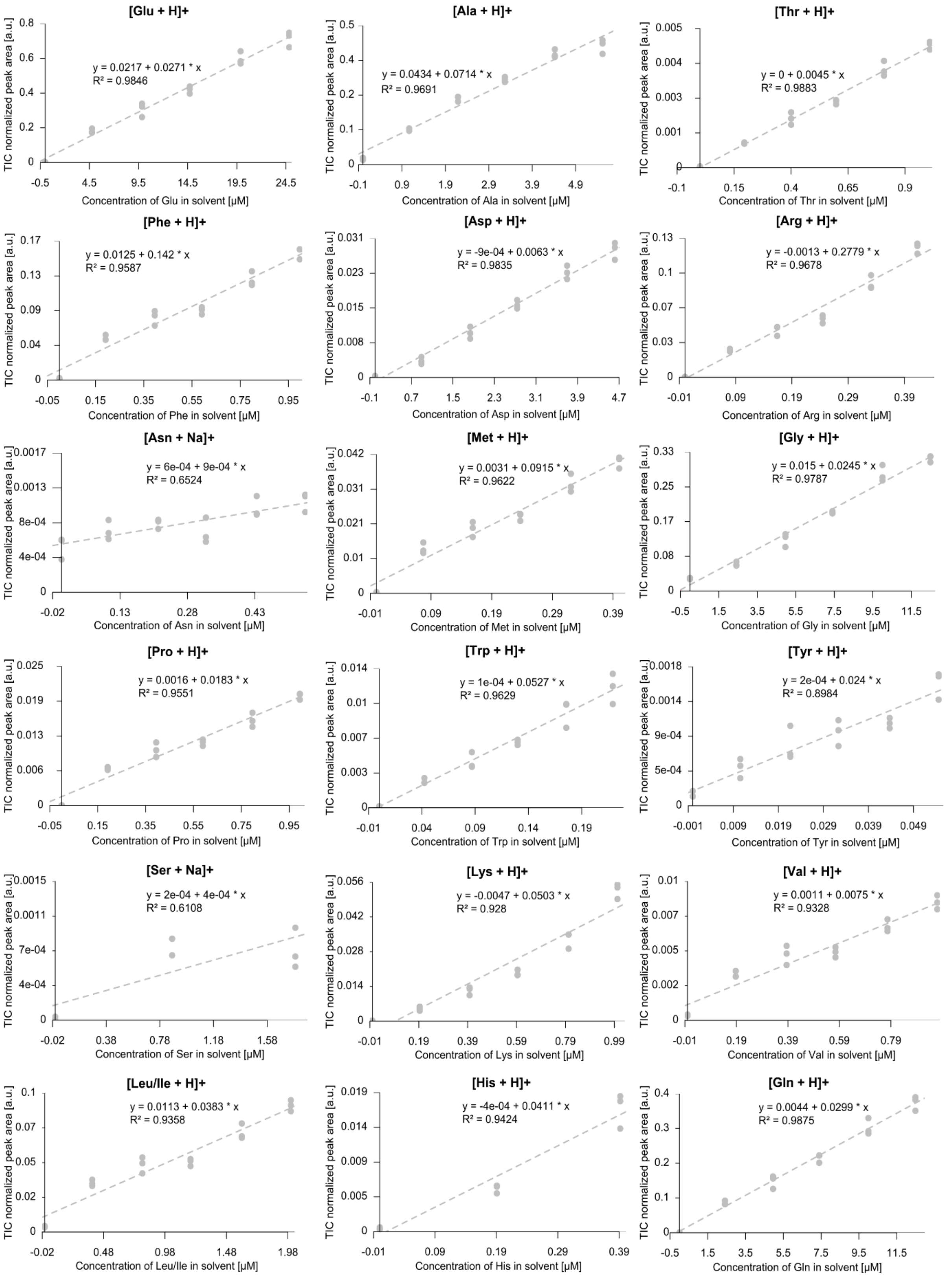

Figure S4. qEC regression curves for amino acids using standards. For amino acid standard response that was not linear over the entire range of concentrations, only the blank concentration and the two lowest concentrations are plotted.

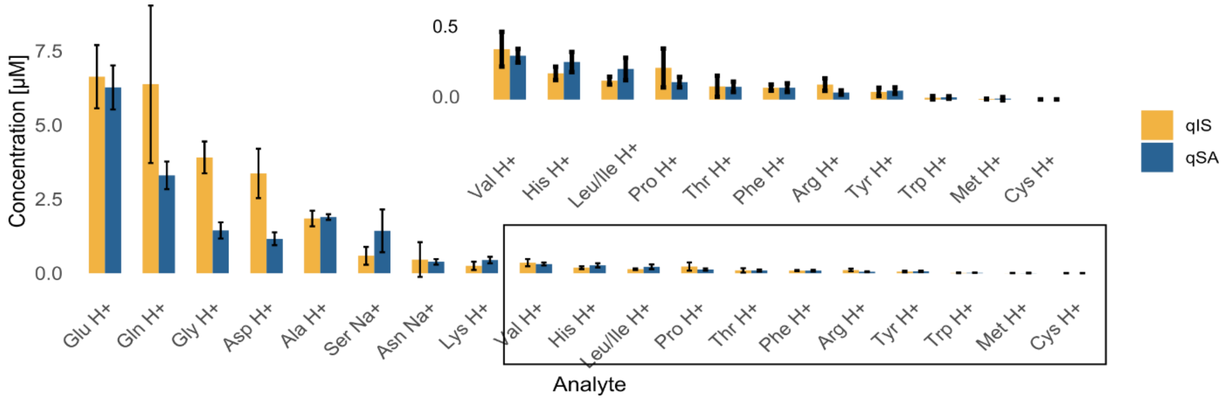

Figure S5. Bar graphs showing the calculated detected AA concentration in brain based on touchdown analysis using qIS (yellow) and qSA (blue). Standard deviations for qIS are derived from triplicate measurements, for qSA from Equation S2. The insert shows enlarged data for low abundant amino acids

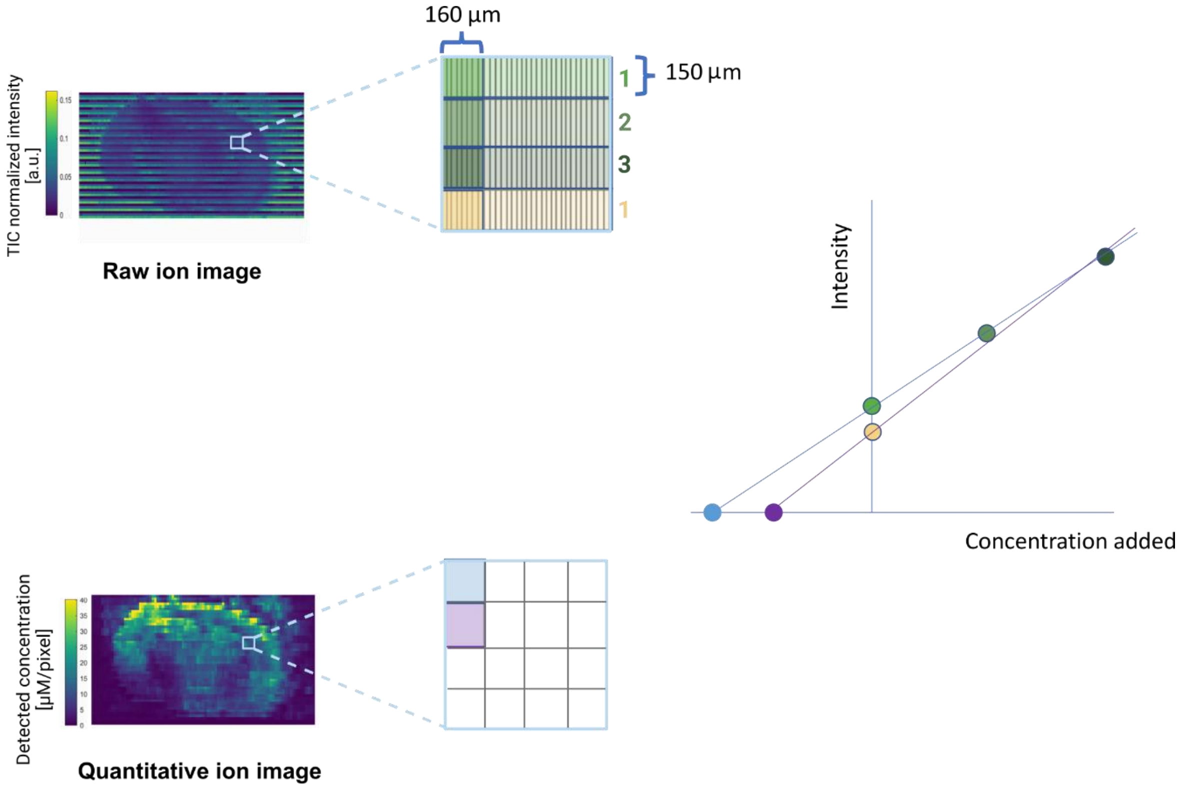

Figure S6. Schematic explanation of the data work flow with a moving reading frame that is used to generate qSA based quantitative ion images. First, 8 pixels are block-averaged in the x-dimension and plotted as a point in the graph. Following, 8 pixels in the subsequent y-line are block-averaged in the x-dimension and plotted as a point in the graph. Finally, 8 pixels in the third y-line are block-averaged in the x-dimension and plotted as a point in the graph. The three points together for the qSA regression curve. The regression is extrapolated to obtain detected concentration, which is input into a pixel of a quantitative ion image. The reading frame moves by one line scan at a time, permitting to keep the spatial resolution in y-dimension intact. Final spatial resolution is thus 160 × 150 µm (X × Y).

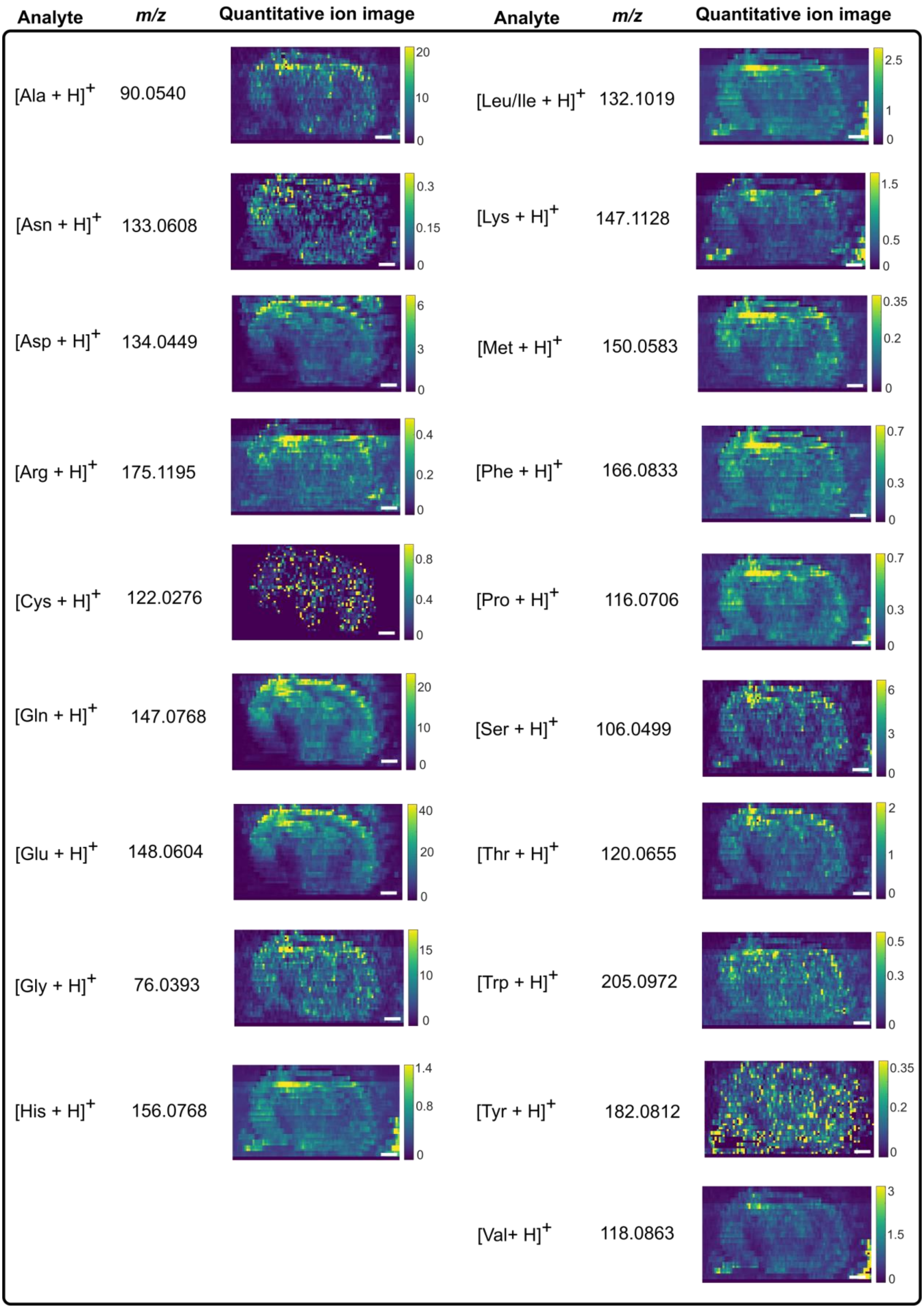

Figure S7. Quantitative ion images of amino acids in a mouse brain tissue section generated with qSA. The colorbar represents detected concentrations [μM/pixel]. The scale bar in right bottom corner corresponds to 1 mm.

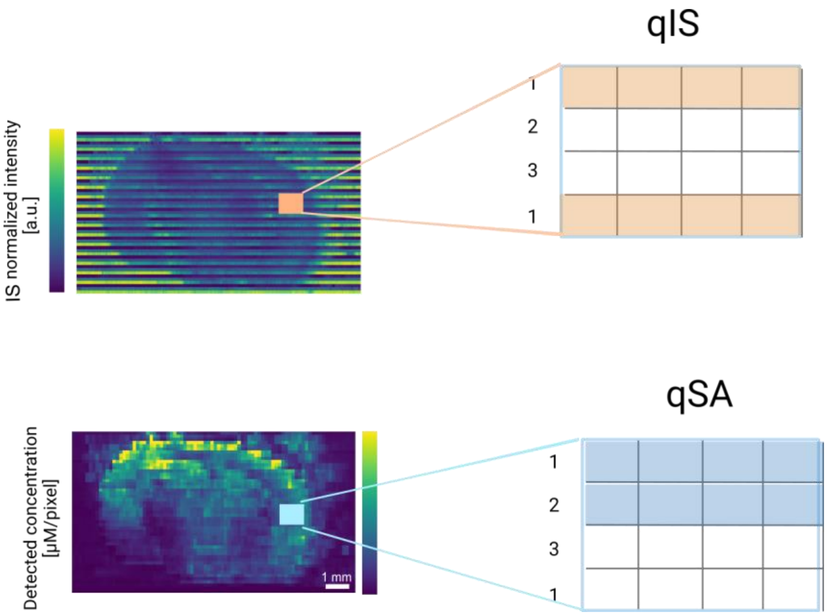

Figure S8. Region of interest (ROI) definition. A matrix of 4×4 pixels is used for each ROI. For qIS, data from two lines with null addition of standards are used, extracted from an IS normalized raw image prior to qSA processing. For qSA, two lines are used within the same ROI after raw image is converted into a qSA image.

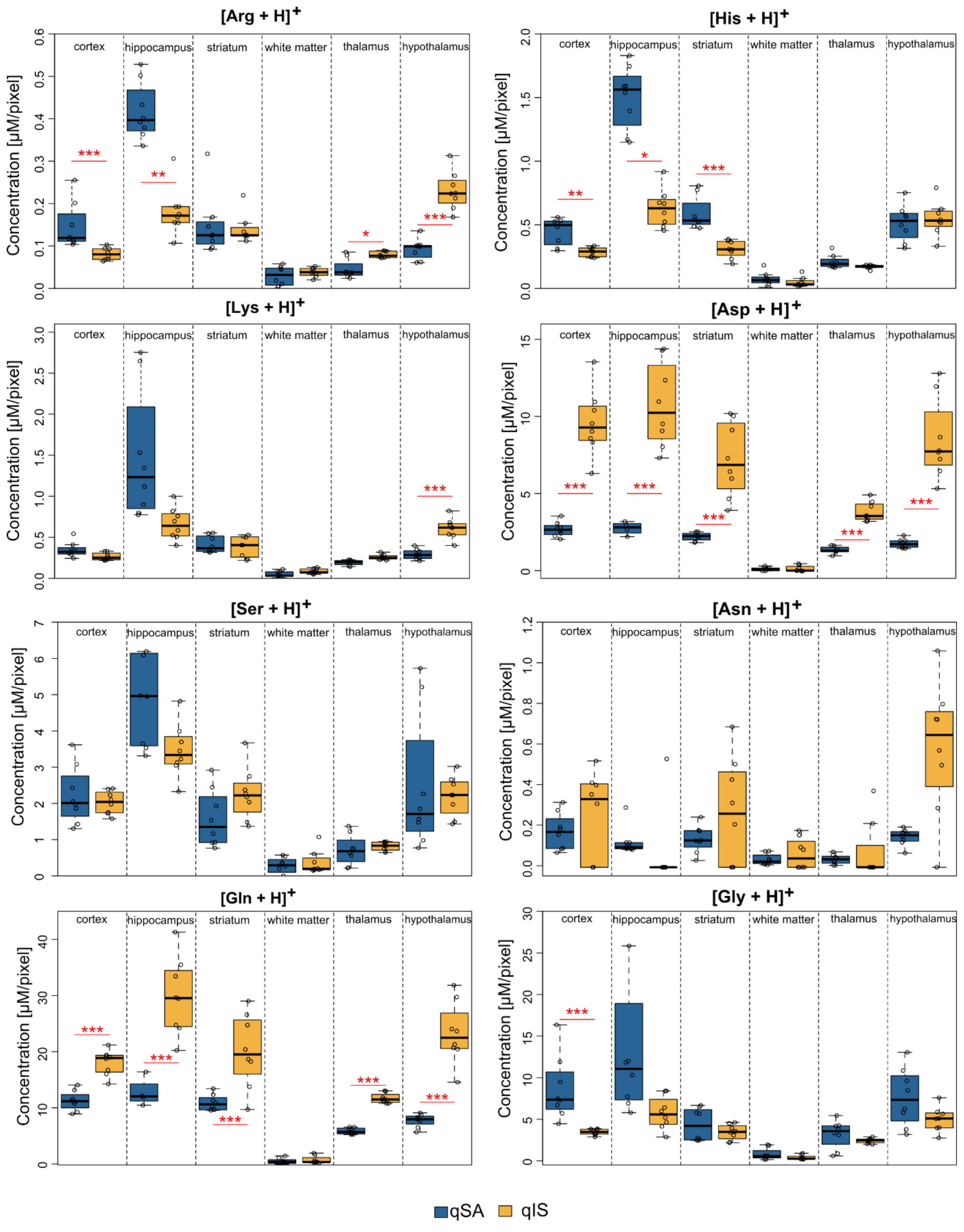
[Arg + H]+
[His + H]+
[Lys + H]+
[Asp + H]+
[Ser + H]+
[Asn + H]+
[Gln + H]+
[Gly + H]+
Concentration [µM/pixel]
cortex
hippocampus
striatum
white matter
thalamus
hypothalamus
qSA
qIS

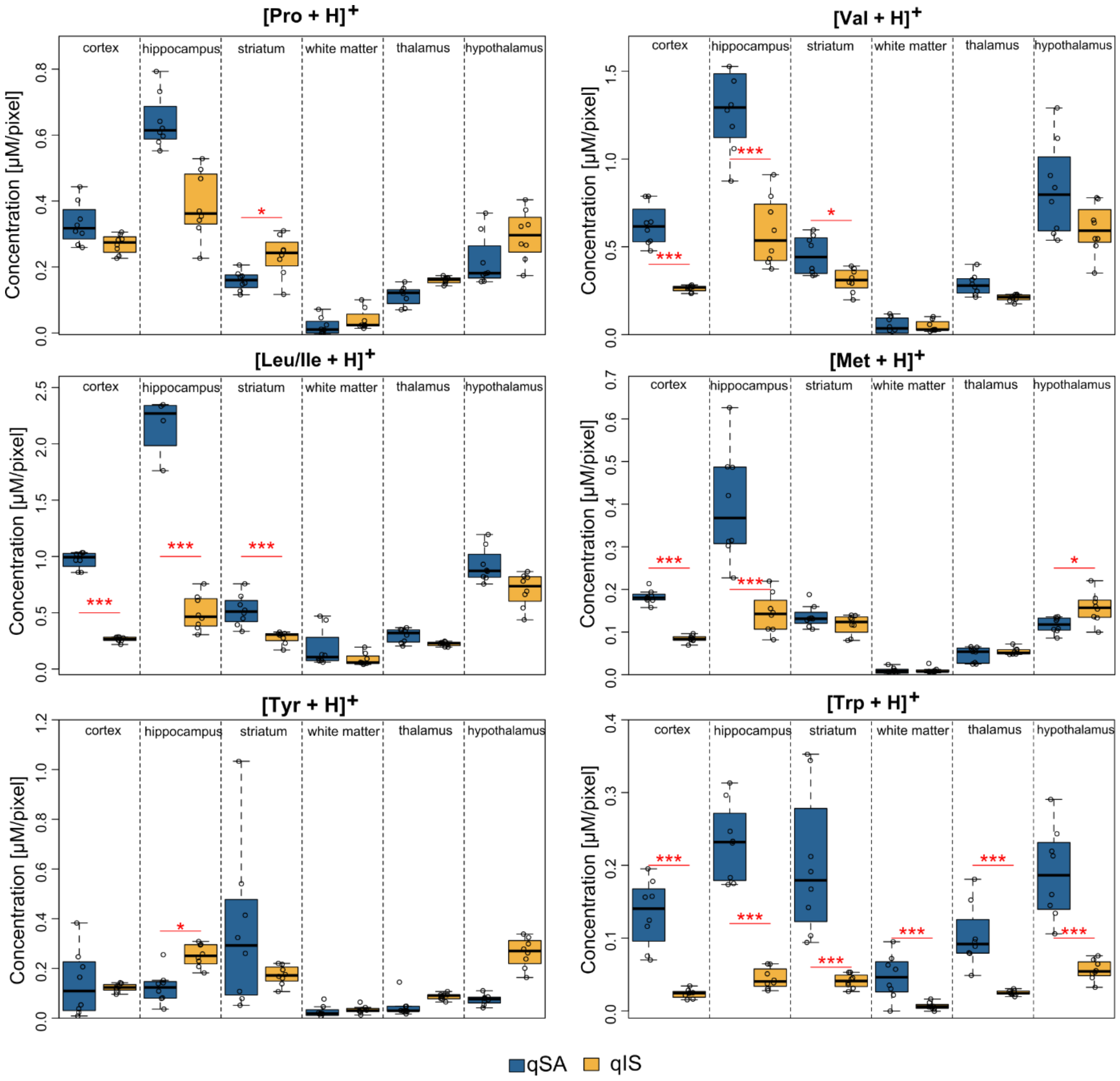

Figure S9. Graphs detailing the detected concentrations of amino acids in 6 different regions of interest using qSA (blue) and qIS (yellow), based on solvents including amino acid standards.

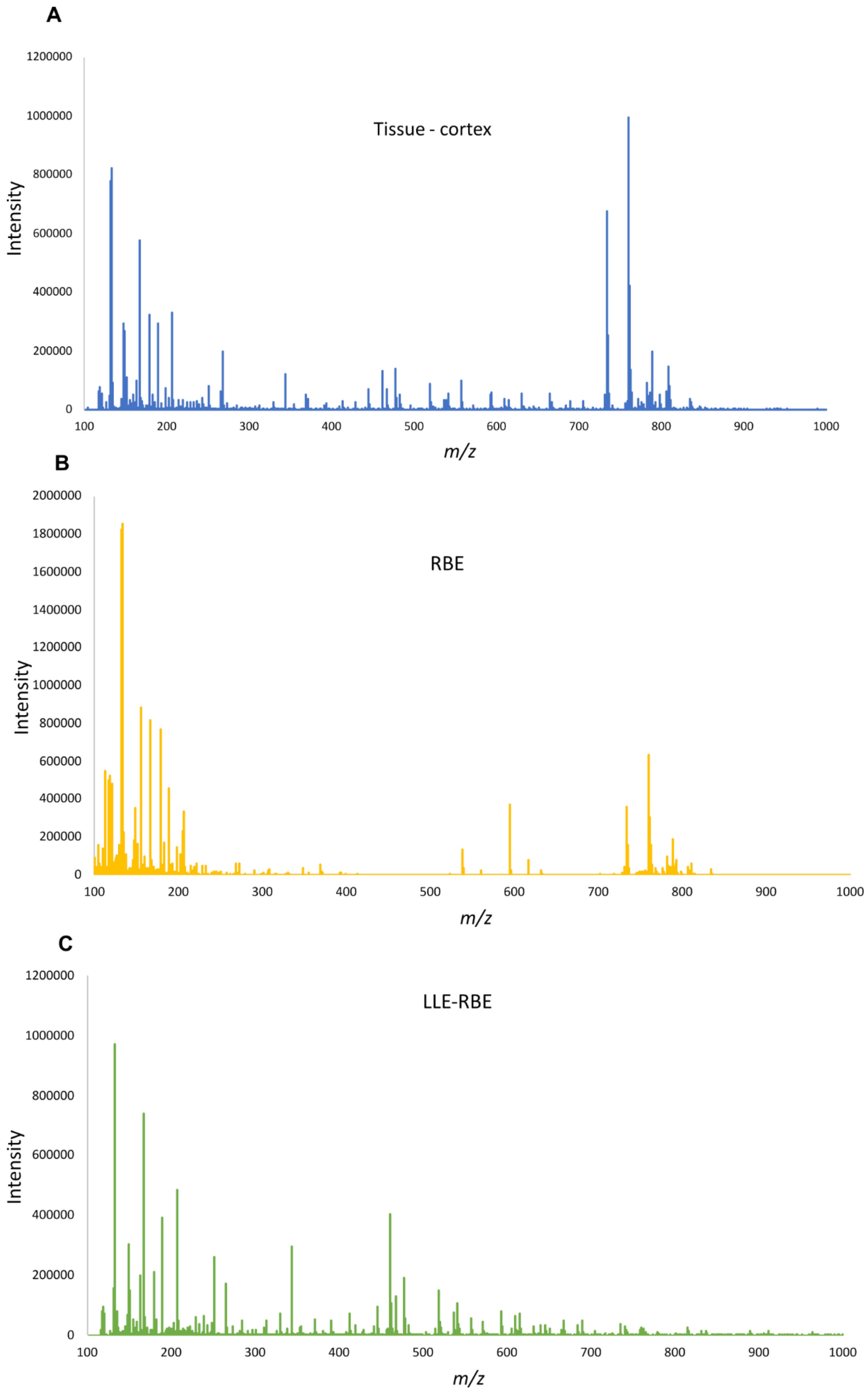

Figure S10. Mass spectra obtained from (A) tissue cortex, (B) RBE, (C) LLE-RBE. For each spectrum presented here, 8 spectra were averaged.

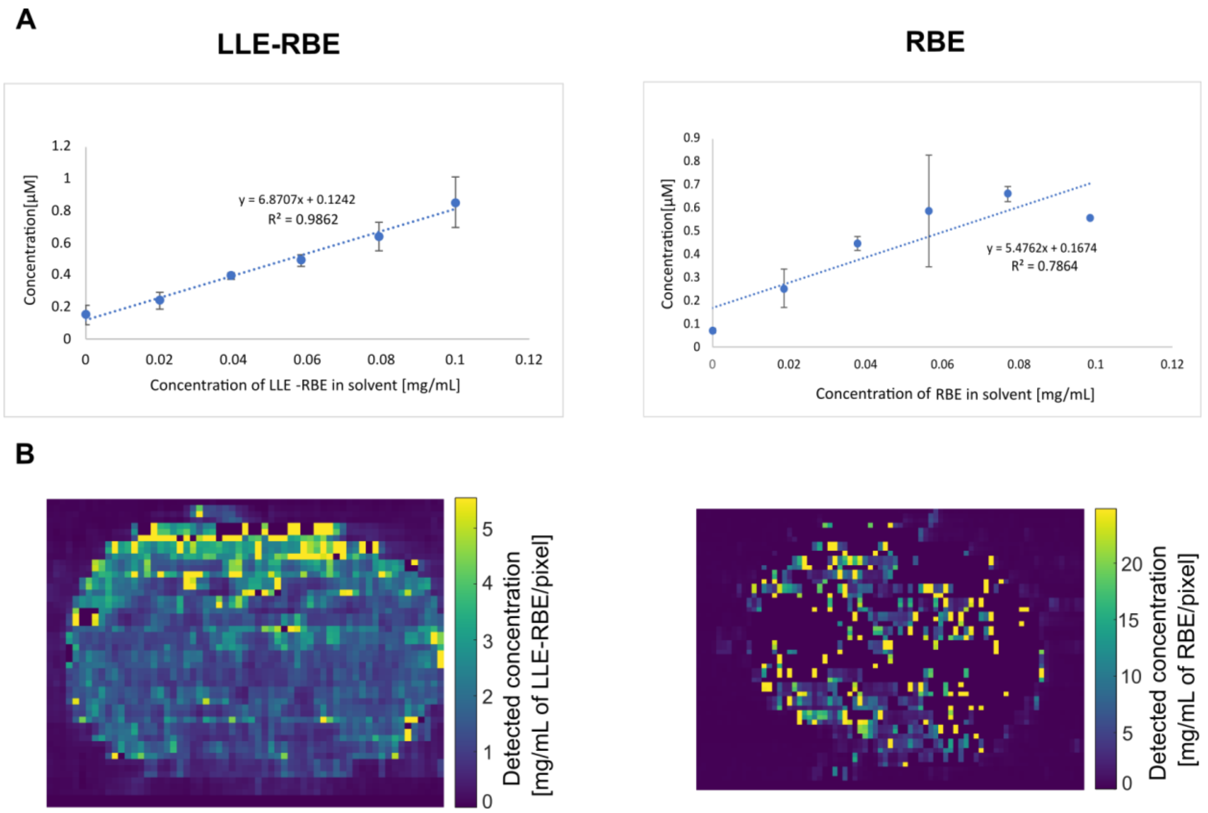

Figure S11. Data for [Glu + H]$^+$ showing (A) qSA regression lines from touchdowns, and (B) quantitative ion images from Q-MSI with standard mixtures containing RBE or LLE-RBE.

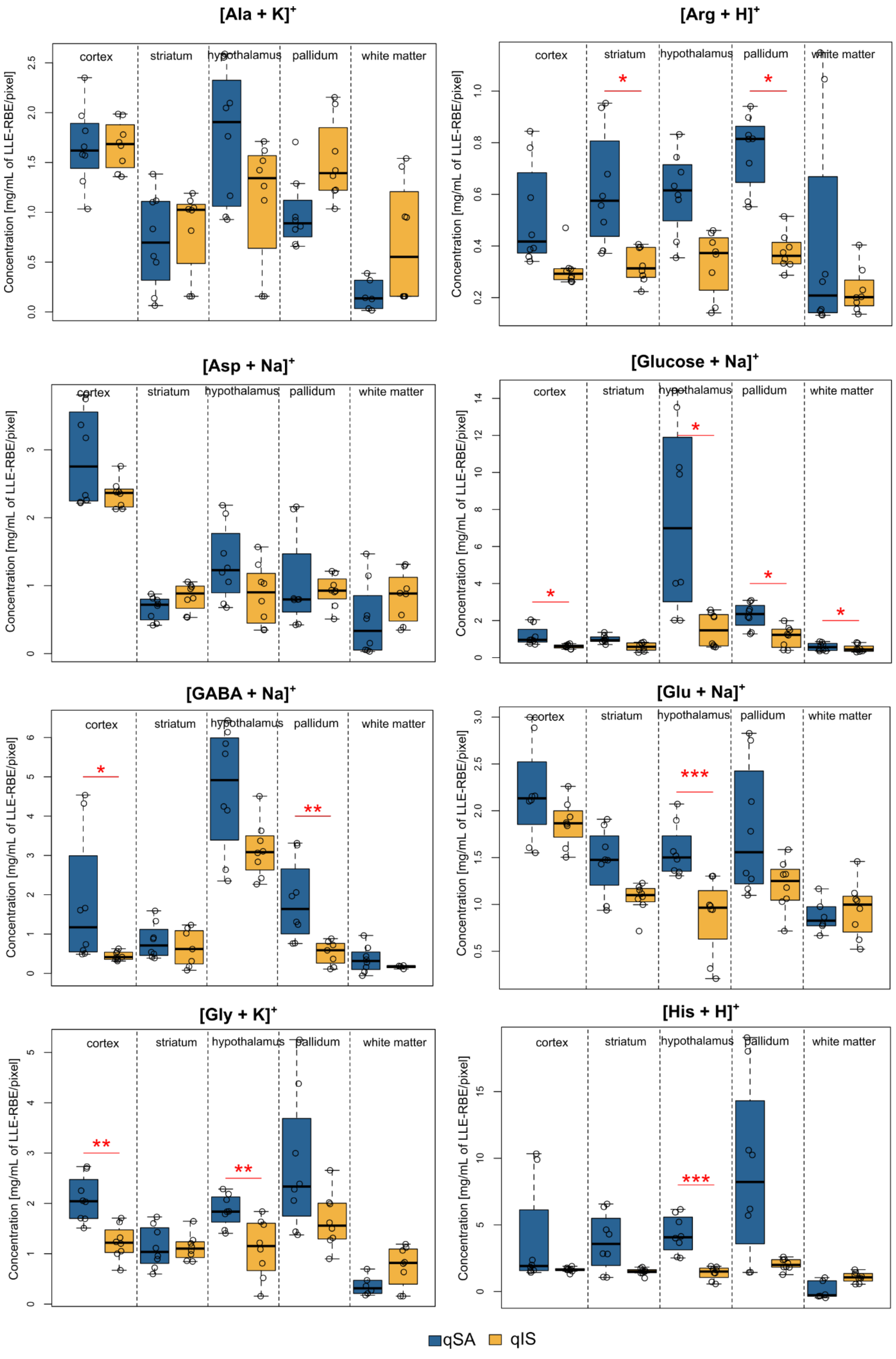
[Ala + K]+
[Arg + H]+
[Asp + Na]+
[Glucose + Na]+
[GABA + Na]+
[Glu + Na]+
[Gly + K]+
[His + H]+
cortex
striatum
hypothalamus
pallidum
white matter
Concentration [mg/mL of LLE-RBE/pixel]
qSA
qIS

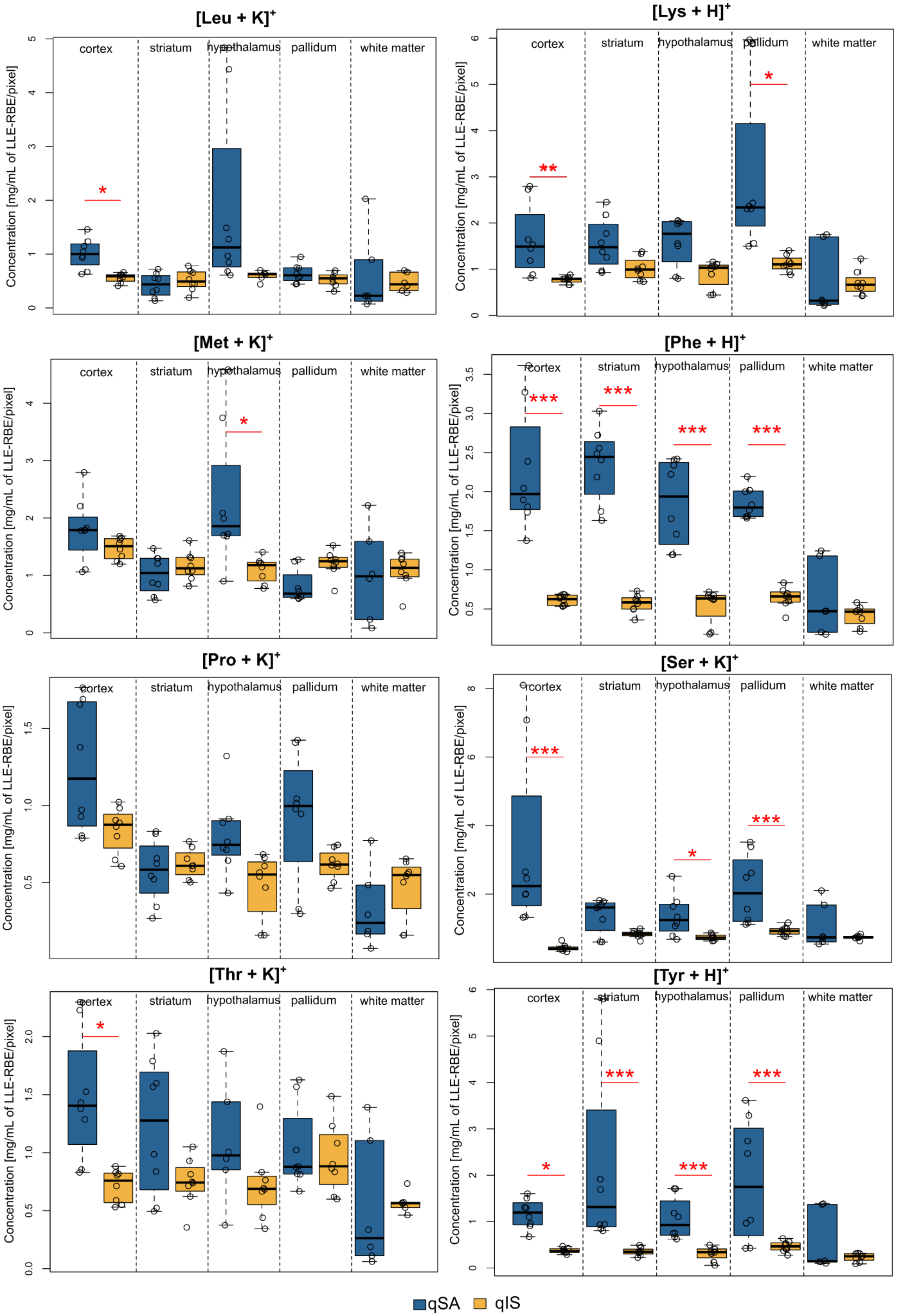
[Leu + K]+
[Lys + H]+
[Met + K]+
[Phe + H]+
[Pro + K]+
[Ser + K]+
[Thr + K]+
[Tyr + H]+
Concentration [mg/mL of LLE-RBE/pixel]
cortex
striatum
hypothalamus
pallidum
white matter
qSA
qIS

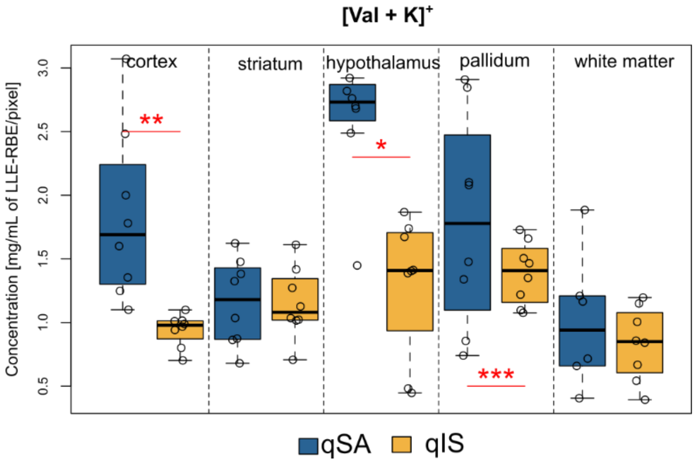

Figure S12. Graphs detailing the detected concentrations of amino acids in 6 different regions of interest using qSA (blue) and qIS (yellow), based on solvents including LLE-RBE. Data is shown for analytes for which an IS was present during MSI.

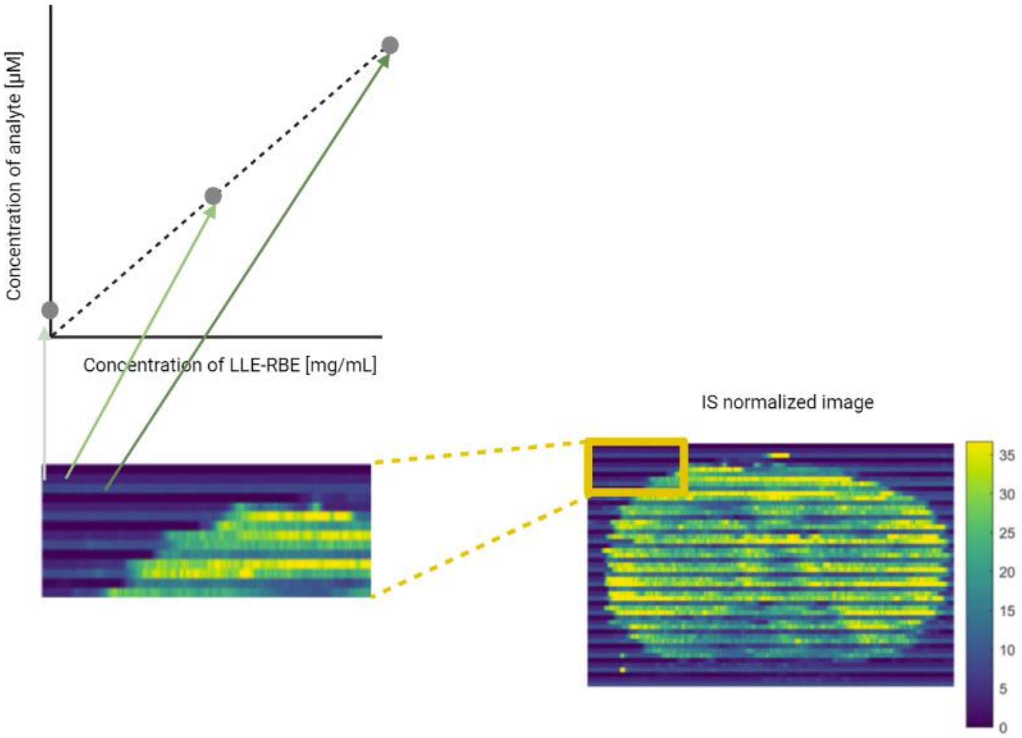

Figure S13. Conversion strategy from LLE-RBE [mg/mL] to concentration [µM] when the concentration of the analyte to be quantified is known in the LLE-RBE. Here, the concentration of the analyte in the LLE-RBE is based on quantification to the IS.

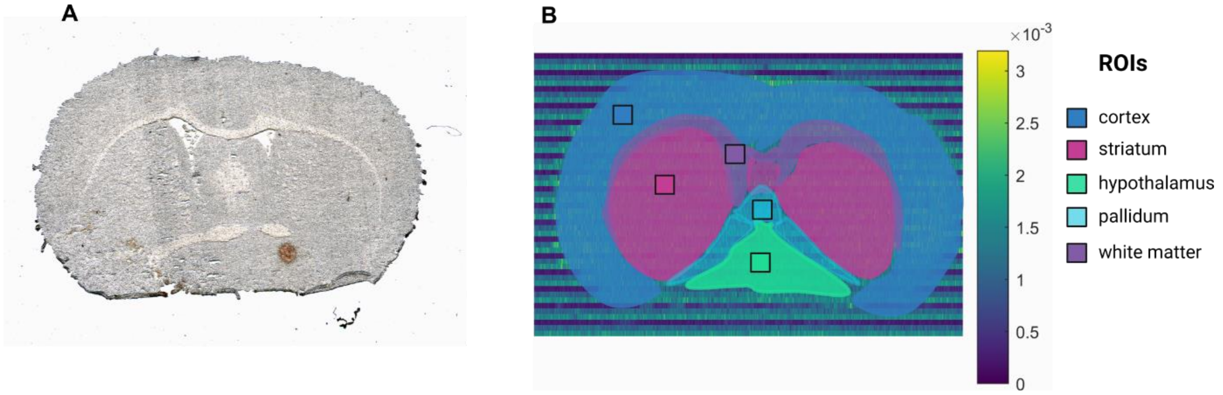

Figure S14. Selected regions of interest (ROIs) for Q-MSI using LLE-RBE for qSA. (A) optical image, (B) 5 regions on mouse brain tissue with defined color coded ROIs.

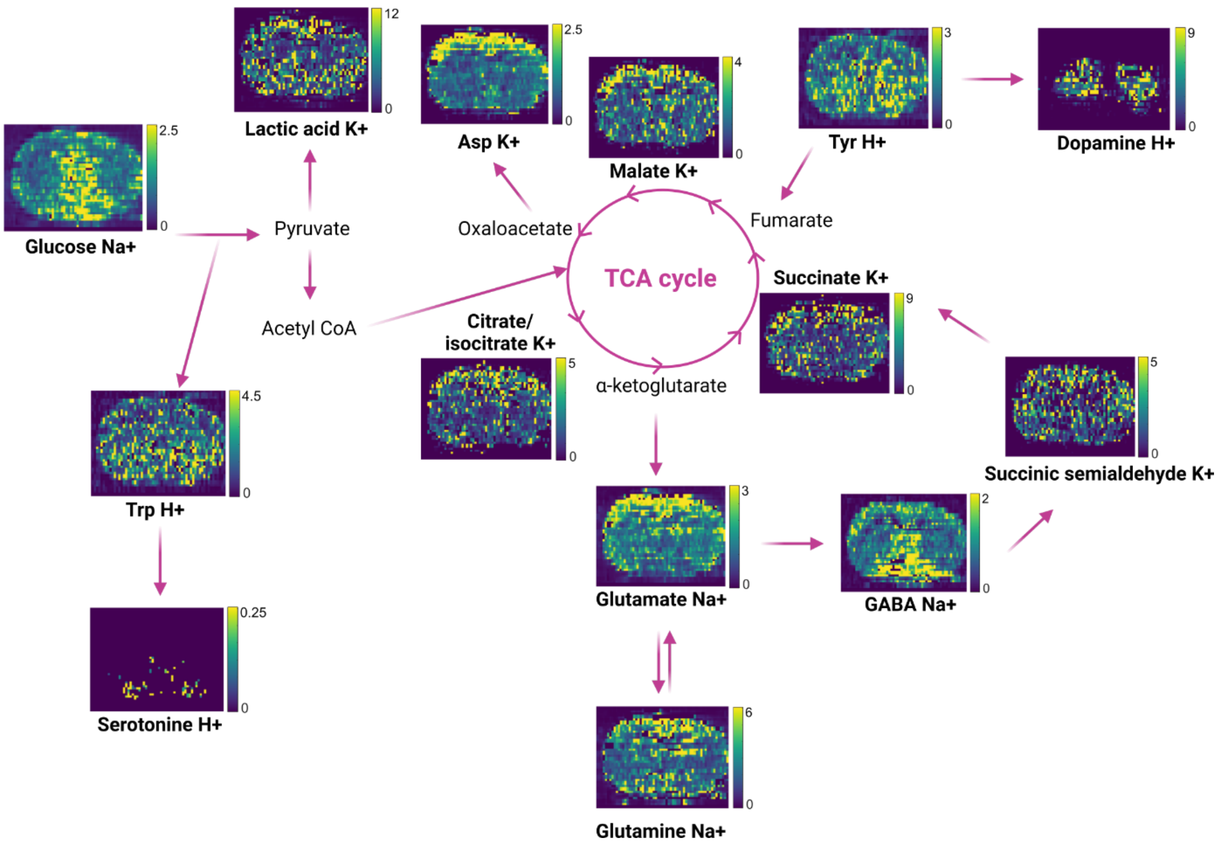

Figure S15. An example of quantitative ion images in a simplified central carbon metabolism scheme with using the non-targeted approach where LLE-RBE is used for qSA. Metabolites are assigned based on exact mass. The detected concentration depicted in the color scale is relative to the concentration of LLE-RBE in the extraction solvent [mg/mL of LLE-RBE/pixel].

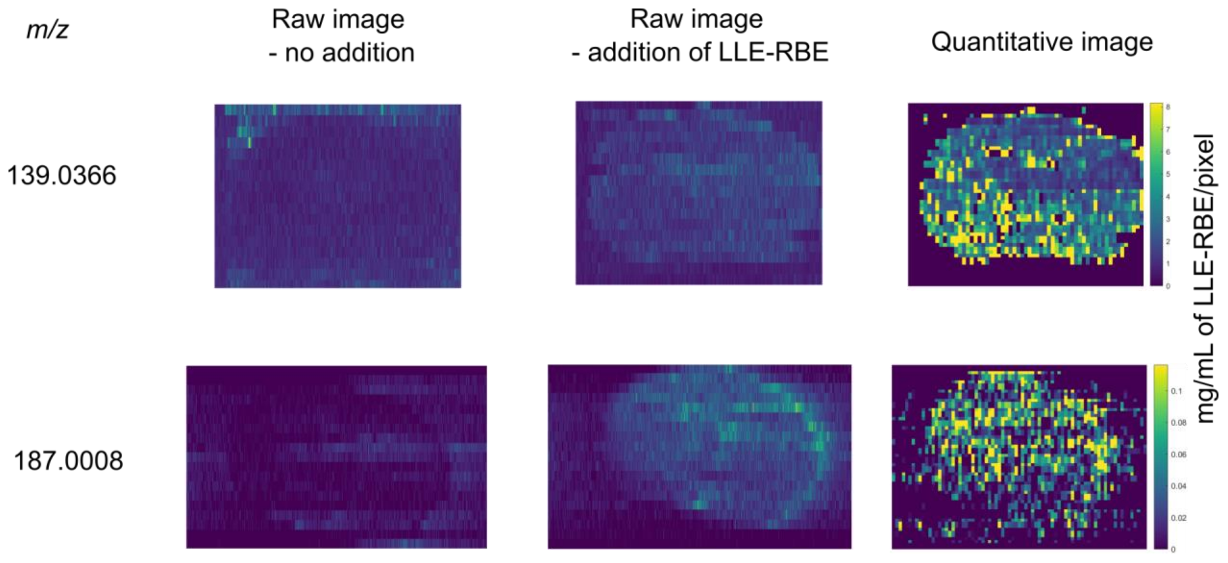

Figure S16. The addition of standards can increase the endogenous analyte signal above the limit of detection. Examples of raw images are shown where no addition of standards was performed (left), and with an addition of LLE-RBE (middle). Although almost undetectable in a classical setup, with qSA the signal intensity for these analytes increases above the limit of detection and a quantitative ion image can be generated (right).

Tables

Table S1: Concentrations in µM of each non-labeled amino acid in calibration solvents (1-6). The concentrations calculated with qSA are given together with the standard deviation and coefficient of determination. The qIS-derived concentration is given together with the standard deviation. The ratio of IS spiked/end conc expresses how close in concentration the endogenous concentration and the IS spiked are. Concentrations derived from qEC are given together with standard deviation and coefficient of determination.

Solvent/ Analyte	1	2	3	4	5	6	qSA	Sd_{qSA}	R^2	qIS	Sd_{qIS}	IS spiked / End conc	qEC	Sd_{qEC}	R^2
Ala	0	1.066	2.191	3.270	4.420	5.520	1.893	0.096	0.9861	1.844	0.263	2.95	0.693	0.007	0.9691
Arg	0.000	0.080	0.164	0.245	0.331	0.413	0.050	0.015	0.9208	0.105	0.045	10.41	0.054	0.026	0.9678
Asn	0.000	0.105	0.215	0.321	0.434	0.542	0.382	0.094	0.5478	0.459	0.586	13.33	0.044	0.009	0.6524
Asp	0.000	0.892	1.833	2.737	3.699	4.619	1.161	0.216	0.8945	3.365	0.833	1.82	1.263	0.409	0.9835
Cys	0.000	0.010	0.021	0.032	0.043	0.054	ND	ND	ND	ND	ND	-	ND	ND	ND
Gln	0.000	2.414	4.958	7.402	10.005	12.493	3.301	0.466	0.9319	6.373	2.658	0.23	2.504	0.891	0.9846
Glu	0.000	4.797	9.854	14.711	19.884	24.829	6.268	0.742	0.9543	6.628	1.067	0.53	3.735	0.155	0.9846
Gly	0.000	2.413	4.957	7.401	10.003	12.490	1.441	0.273	0.9688	3.907	0.537	1.08	1.007	0.080	0.9787
His	0.000	0.191	0.392	0.585	0.790	0.987	0.264	0.071	0.7602	0.184	0.047	1.98	0.144	0.071	0.9424
Leu/Ile	0.000	0.389	0.798	1.192	1.611	2.012	0.215	0.079	0.9039	0.133	0.027	39.98	ND	ND	0.9358
Lys	0.000	0.194	0.398	0.594	0.803	1.003	0.443	0.109	0.6747	0.247	0.140	5.01	0.251	0.143	0.9280
Met	0.000	0.077	0.159	0.237	0.320	0.400	0.006	0.011	0.9421	0.003	0.002	194.30	ND	ND	0.9622
Phe	0.000	0.193	0.396	0.591	0.798	0.997	0.084	0.030	0.9391	0.083	0.022	17.55	ND	ND	0.9587
Pro	0.000	0.192	0.395	0.589	0.796	0.994	0.123	0.036	0.9177	0.223	0.137	6.53	ND	ND	0.9551
Ser	0.000	0.872	1.790	2.673	3.613	4.511	1.427	0.722	0.5954	0.586	0.301	3.60	ND	ND	0.6108
Thr	0.000	0.195	0.400	0.598	0.808	1.009	0.089	0.036	0.9171	0.093	0.074	25.85	0.102	0.084	0.9883
Trp	0.000	0.042	0.087	0.130	0.176	0.219	0.014	0.008	0.9037	0.012	0.012	121.40	0.007	0.002	0.9629
Tyr	0.000	0.011	0.022	0.032	0.044	0.055	0.063	0.023	0.2646	0.054	0.029	13.48	0.039	0.022	0.8984
Val	0.000	0.187	0.385	0.575	0.777	0.970	0.309	0.048	0.8931	0.355	0.122	7.38	0.101	0.079	0.9328

Table S2: Concentrations in mg/mL of LLE-RBE and RBE in calibration solvents (1-6). Solvents used for MSI experiments are shaded in gray.

Solvent	LLE-RBE	RBE
1	0	0
2	0.020	0.019
3	0.039	0.039
4	0.058	0.055
5	0.460	0.467
6	0.954	0.946

References

(1) Harris, D. C.; Lucy, C. A. *Quantitative chemical analysis*; W. H. Freeman and Company, 2016.
(2) Bergman, H.-M.; Lundin, E.; Andersson, M.; Lanekoff, I. Quantitative mass spectrometry imaging of small-molecule neurotransmitters in rat brain tissue sections using nanospray desorption electrospray ionization. *Analyst* **2016**, *141* (12), 3686-3695, DOI: 10.1039/C5AN02620B.
